# Optimization of epilepsy surgery through virtual resections on individual structural brain networks

**DOI:** 10.1038/s41598-021-98046-0

**Published:** 2021-09-24

**Authors:** Ida A. Nissen, Ana P. Millán, Cornelis J. Stam, Elisabeth C. W. van Straaten, Linda Douw, Petra J. W. Pouwels, Sander Idema, Johannes C. Baayen, Demetrios Velis, Piet Van Mieghem, Arjan Hillebrand

**Affiliations:** 1 Department of Clinical Neurophysiology and MEG Center, Amsterdam Neuroscience, Vrije Universiteit Amsterdam, Amsterdam UMC, Amsterdam, The Netherlands; 2 Department of Anatomy and Neuroscience, Amsterdam Neuroscience, Vrije Universiteit Amsterdam, Amsterdam UMC, Amsterdam, The Netherlands; 3 Radiology and Nuclear Medicine, Amsterdam Neuroscience, Vrije Universiteit Amsterdam, Amsterdam UMC, Amsterdam, The Netherlands; 4 Department of Neurosurgery, Amsterdam Neuroscience, Vrije Universiteit Amsterdam, Amsterdam UMC, Amsterdam, The Netherlands; 5 Faculty of Electrical Engineering, Mathematics and Computer Science, Delft University of Technology, Delft, The Netherlands

**Keywords:** Epilepsy, Computational neuroscience, Network models

## Abstract

The success of epilepsy surgery in patients with refractory epilepsy depends upon correct identification of the epileptogenic zone (EZ) and an optimal choice of the resection area. In this study we developed individualized computational models based upon structural brain networks to explore the impact of different virtual resections on the propagation of seizures. The propagation of seizures was modelled as an epidemic process [susceptible-infected-recovered (SIR) model] on individual structural networks derived from presurgical diffusion tensor imaging in 19 patients. The candidate connections for the virtual resection were all connections from the clinically hypothesized EZ, from which the seizures were modelled to start, to other brain areas. As a computationally feasible surrogate for the SIR model, we also removed the connections that maximally reduced the eigenvector centrality (EC) (large values indicate network hubs) of the hypothesized EZ, with a large reduction meaning a large effect. The optimal combination of connections to be removed for a maximal effect were found using simulated annealing. For comparison, the same number of connections were removed randomly, or based on measures that quantify the importance of a node or connection within the network. We found that 90% of the effect (defined as reduction of EC of the hypothesized EZ) could already be obtained by removing substantially less than 90% of the connections. Thus, a smaller, optimized, virtual resection achieved almost the same effect as the actual surgery yet at a considerably smaller cost, sparing on average 27.49% (standard deviation: 4.65%) of the connections. Furthermore, the maximally effective connections linked the hypothesized EZ to hubs. Finally, the optimized resection was equally or more effective than removal based on structural network characteristics both regarding reducing the EC of the hypothesized EZ and seizure spreading. The approach of using reduced EC as a surrogate for simulating seizure propagation can suggest more restrictive resection strategies, whilst obtaining an almost optimal effect on reducing seizure propagation, by taking into account the unique topology of individual structural brain networks of patients.

## Introduction

Resective epilepsy surgery is the treatment of choice for refractory epilepsy in carefully selected patients who undergo a rigorous presurgical evaluation^1^. Often, patients with a good surgical outcome are not only free of disabling seizures, but also experience an improvement in their cognitive abilities and quality of life^2^. Even though most patients achieve an improvement in the seizure burden, epilepsy surgery is not always successful and may require repeat surgery^3^. Approximately one-third of patients do not become seizure-free after surgery^4^. The success of surgery depends on successful localization of the epileptogenic zone and the choice of the resection area. In the last decade, a rethinking of the concept of the epileptogenic zone has taken place. The epileptogenic zone is defined as a circumscribed area that needs to be removed or disconnected to achieve seizure freedom^5^. However, current views advocate the existence of an epileptogenic network instead of a locally confined epileptogenic zone^6–10^. In a network, local resections can have widespread effects, even at remote locations, that cannot be directly predicted from the location of the removed region itself^11,12^. Similarly, a local resection of pathological tissue might not prevent the epileptogenic network from forming a new seizure onset zone (defined as the regions where seizures start^5^) over time^13^. The brain network varies from one patient to the next^14,15^, but the network itself and its variability between patients are not taken into account in current surgical procedures. To find the area in a network that should be resected requires imaging modalities that cover the entire brain, as well as ways to evaluate the effect of a local resection on activity in the entire network at the level of the individual patient (precision medicine).

Side-effects and cognitive complaints often occur after surgery, depending on the brain areas involved, but these complaints vary between patients and are difficult to predict^4^. Some of the side-effects and complaints could potentially be avoided with a smaller resection area. Resection areas are often larger than necessary^16^, sometimes because healthy tissue is removed to access the pathologic tissue (as in mesial temporal resections), but also because the risk of not removing all of the pathological tissue, resulting in recurrent seizures, often outweighs the benefits of fewer side-effects after surgery^17^. A priori tailoring of resection areas would be feasible if different resection strategies could be tested beforehand and network effects predicted reliably.

Individualized computer models to aid epilepsy surgery could overcome many of the above mentioned challenges and thereby improve surgical outcome^18–20^. The main advantages are (1) surgery can be individualized by modelling activity, and in particular the propagation of seizures, on a patient-specific structural brain network, and (2) competing resection strategies can be tested in silico before the actual surgery, thereby demonstrating which strategy would be the most effective at the lowest ‘cost’ in terms of removed tissue. The individualization of the model can be based on some important interpersonal differences for epilepsy: the location of the seizure onset zone (SOZ) and the pathways of propagation of seizure activity. The propagation strongly depends on the structural brain network of the patient^21^, which maps the anatomical connections between the different brain regions. It is conceivable that in many patients resections can be smaller than is currently the case, as the model can test which tissue/connection needs to be removed and which can safely remain in place whilst still achieving a satisfactory control of seizures. In addition, more effective resections can also be searched for, for example at different locations or when there are multiple hypotheses about the EZ location.

Several recent studies have applied computer models to smaller groups of patients with epilepsy^22–32^. Most model studies identify epileptogenic areas using their model and predict surgery outcome^25,26,29,32–34^. For example, both Sinha et al.^29^ and Goodfellow et al.^33^ found epileptogenic areas in their model, virtually resected them, and predicted surgery outcome based on the effect on the model with an accuracy of 81%^29^ or an area-under-the-curve (AUC) of 0.87^33^. Another study calculated the overlap of the epileptogenic nodes identified by the model with the actual resection and found larger overlap in patients with a good outcome^24^. In a recent study, Sip et al.^32^ modelled the propagation of ictal activity (as recorded via invasive EEG) on patient-specific structural networks, and found a link between the excitable regions inferred by the model and the clinically found epileptogenic zone^32^. In ictal functional networks, the elevated ability to generate seizures^26^ as well as the fraction of virtually resected rich-club nodes^25^ correlated with surgery success. Other studies used computer models to find epileptogenic nodes and evaluated their properties. The virtual resection of those epileptogenic nodes reduced the epileptogenic activity^31^, and had a larger effect compared to a standard clinical resection in temporal lobe epilepsy (TLE) patients^23^.

Another approach is to test the actual surgery in the model: Steimer et al.^30^ removed the areas that were resected during the actual surgery in their model and found that the developing seizure could be stopped in seizure-free (SF) but not in not seizure-free (NSF) patients^30^. Olmi and colleagues^27^ used the actually resected connections as reference and showed that removing fewer connections in their model achieved the same effect as the actual surgery^27^. Thus, instead of removing the EZ, the seizure propagation pathways were severed to ensure seizure freedom. The same group compared the areas of propagation in the model to those in SEEG and concluded that the overlap predicted surgery outcome^28^. Removing the propagation pathways to limit seizure propagation is an effective approach in cases where the epileptogenic zone involves eloquent cortex and therefore would be inoperable^22^.

These studies have used different models (see Junges et al.^18^ for detailed description): models based on neural masses^22,27,28,31,33^, theta models—an approximation of neural masses in networks^24–26^, bistable models^23,29,35^, and a distributional clustering model^30^. All these models are complicated with several (often nonlinear) parameters that need to be estimated beforehand. This hampers the accurate estimation of the optimal resection strategy, as every parameter needs to be adjusted to the optimal range in order to give an accurate and reproducible prediction for each tested resection strategy. Simpler models with few parameters might prove more reproducible, especially if the behavior of the model is understood mathematically. A full mathematical understanding of a model aids the application and especially the interpretation of the results.

In a model, the brain activity is typically simulated on the backbone of a network^23–29, 33^. Such a network can be derived for each patient from functional^24–26,29,33^ or structural^22,23,27,28^ data. However, as the function (brain activity) is simulated using the model, it is more intuitive to use a structural network on which the dynamics take place. Some of the previous studies have used whole brain networks^22,23, 27, 28^, whereas others have studied partial networks derived from intracranial recordings^24–26,29,33^. The limitation of partial networks is that network-wide effects beyond the assessed brain areas are not taken into account. It is therefore preferable to study structural and entire brain networks, as they map the main pathways of all lobes of the brain upon which the functional activity unfolds.

A recent study performed virtual resections based on a network framework, such as our surrogate approach^36^. Their measure to estimate the effect of the virtual surgery was synchronizability, which is a measure of the ability to maintain global synchronization^37^. The authors calculated synchronizability on functional networks derived from electrocorticography (ECoG) measurements and found that the change in synchronizability after virtually removing the resection area predicted surgical outcome, although this result was not significant after multiple comparisons corrections. However, synchronizability is defined for structural networks and requires several assumptions that are not fulfilled in brain data, such as identical dynamical units^38^. Its application to functional brain networks leaves the interpretation unclear and without theoretical foundation.

Here we introduce a personalized computer model, based upon individual structural brain networks, to guide epilepsy surgery and improve surgical outcome. The dynamic model is derived from the field of epidemics and has the advantage that it is mathematically well-understood, simple and only has two free parameters^39,40^. The model captures one aspect of the epileptic brain that is relevant for surgery, namely the propagation of seizures over the network, rather than the underlying biology. We chose an epidemic model to simulate this process, because there is a striking similarity in the way an infectious disease spreads in a population and how a seizure spreads in a brain network. Furthermore, many definite results have been obtained in the study of spreading of infections on graphs^40^. This knowledge can be used to guide model choice and interpretation. We applied this model to the whole-brain network derived from diffusion tensor imaging (DTI) for each individual patient, thereby personalizing the optimal virtual resection. The model was tested retrospectively in a preliminary study with 19 patients who had been operated, and of whom the surgical outcome at least 1 year after surgery was known.

## Methods

### Patients

We retrospectively analyzed 19 patients (11 females) with refractory epilepsy (Table 1). All patients underwent epilepsy surgery at the Amsterdam University Medical Center, location VUmc, between 2013 and 2016. We included all patients who for their presurgical evaluation had received a clinical DTI scan and magnetoencephalography (MEG) recording since 2010 (the MEG data was not used in this study). We excluded patients with previous epilepsy surgery, an infiltrating tumor or absence of a signed informed consent. The patient group was heterogeneous with temporal and extratemporal resection locations and different etiology, the most common being mesial temporal sclerosis (MTS), malformations of cortical development (MCD), and gliosis. Surgical outcome was classified according to the Engel classification at least one year after the operation. Patients with Engel class 1 were labelled as SF, and patients with Engel class 2–4 were labelled as NSF. All patients gave written informed consent and the study was in accordance with the Declaration of Helsinki and approved by the VUmc Medical Ethics Committee.

**Table 1:** Patient characteristics of the seizure-free (upper part) and not seizure-free patients (lower part).

Patient no	Age at surgery (years)	Gender	Age at epilepsy onset (years)	Years epilepsy at resection	Resection area	MRI radiologic diagnosis	Pathology results	Engel score	Surgery outcome
1	32	F	4	28	R Front	FCD	FCD; Gliosis	1A	SF
2	20	F	4	17	R Temp	Abnormal WM signal R temporal pole	mMCD; Gliosis	1A	SF
5	25	M	3	22	R Front	Neoplasm—benign tumor	FCD	1A	SF
6	27	F	6	21	R Front	FCD	No classified diagnosis	1A	SF
7	33	F	18	14	R Temp	MTS	MTS	1B	SF
8	49	M	16	33	R Temp	MTS	MTS	1A	SF
9	19	M	11	12	R Temp	MTS	MTS	1A	SF
10	22	F	12	10	R Temp	MTS	MTS	1A	SF
11	25	M	16	8	R Temp	MTS	Gliosis	1A	SF
12	47	F	35	13	L Temp	MTS	MTS	1A	SF
13	54	F	21	33	R Temp	MTS	MTS	1A	SF
14	53	M	47	6	R Temp	Unknown	Gliosis	1A	SF
16	32	F	2	30	R Temp	MTS	MTS	1A	SF
17	24	F	8	17	L Temp	MTS	Cerebral abscess; MTS	1A	SF
3	40	M	35	6	R Temp	Cavernoma	Cavernoma; Gliosis	2B	NSF
4	26	F	11	15	R Temp	Unknown	mMCD	2A	NSF
15	47	M	26	21	R Front	Polymicrogyria	Polymicrogyria; Heterotopy	4B	NSF
18	25	M	18	7	R Temp	MTS	Negative	4A	NSF
19	54	F	23	31	R Temp	MTS	Gliosis	2D	NSF

F = female, M = male, R = right, L = left, Front = frontal, Temp = temporal, FCD = focal cortical dysplasia, WM = white matter, MTS = mesial temporal sclerosis, mMCD = mild malformation of cortical development, SF = seizure-free, NSF = not seizure-free.

### Individualized structural networks

#### DTI and T1 acquisition

The individualized computer model was based on the structural brain network of each patient, which was reconstructed from the patient’s DTI scans. The magnetic resonance imaging (MRI) scans (T1 and DTI) were acquired on a 3T whole-body MR scanner (Discovery MR750, GE Healthcare, Milwaukee, WI, USA) using an eight-channel phased-array head coil. Anatomical 3D T1-weighted images were obtained at 1 mm isotropic resolution with a fast spoiled gradient-recalled echo sequence (repetition time: 4.6 ms, echo time: 2.0 ms, inversion time: 650 ms, flip angle: 15°). The DTI were obtained with a 2D echo-planar sequence (repetition time: 7200 ms, echo time: 80 ms, 70 slices, 2 mm isotropic resolution, 24 diffusion gradient directions (b = 750 s/mm^2^) and 4 non-diffusion-weighted measurements. During reconstruction, images were interpolated to 1 × 1 mm in-plane resolution.

#### DTI and T1 processing

The following processing steps of the MR images were performed using the Functional MRI of the Brain (FMRIB) software library (FSL), version 5.0.10^41^. Structural connectivity matrices were derived from probabilistic tractography between 92 pre-defined regions-of-interest (ROIs). We used 78 cortical ROIs^42^ from the automated anatomical labeling (AAL) atlas^43^ defined in MNI standard space and 14 subcortical ROIs from FIRST^44^ defined in T1-space. The pre-operative T1 scan was registered to the MNI template of the AAL atlas, and the inverse transformation was used to convert the AAL ROIs to T1 subject space. Subsequently, the cortical and subcortical ROIs were combined in T1 space. The rim of each ROI (i.e. the border between grey and white matter) was used as a seed as well as a target point for probabilistic tracking. For the cortical ROIs, the white matter mask was obtained using SIENAX^45^, and a rim of two voxels thickness was obtained by first dilating the white matter mask and subsequently subtracting an eroded white matter mask. The resulting rim was multiplied with the AAL atlas in subject space to get rim masks for each cortical ROI. Rim masks of the subcortical ROIs were calculated by eroding the subcortical masks by two voxels and subtracting this eroded mask from the original subcortical masks. For the DTI images, head motion and eddy currents were corrected using EDDY from FMRIB’s Diffusion Toolbox (FDT). The default of three fiber orientations were modelled per voxel using BEDPOSTX^46^, and probabilistic tracking was done using PROBTRACKX2 in DTI space^46^. From each voxel in the seed ROIS 5000 tracts were started (with a curvature threshold of 0.2 and step length of 0.5 mm) and were terminated if one of the 91 target ROIs was reached. We discarded tracts with interhemispheric crossings outside the corpus callosum or the fornix. The number of tracts between ROIs were not corrected for the size of the seed or target region, as it is realistic that larger regions have more tracks than smaller regions.

#### Structural network

The sum of all tracts from each ROI to all other ROIs resulted in a weighted DTI connectivity matrix. The tract counts were averaged in both directions to get a symmetrical matrix. The matrix was binarized and thresholded to a connection density of 11%, to match the connection density of a literature-based structural matrix obtained from 80 healthy subjects^42^. Additionally, we obtained an average matrix by averaging all individual full weighted matrices and thresholded it to 11% connection density, and compared the main results of using the individual matrices to results using the average matrix. We also compared the main results using the binarized and thresholded matrices to results obtained using the weighted matrix without a threshold.

### SIR model

To simulate the propagation of seizure activity on individualized structural brain networks we used the susceptible-infected-recovered (SIR) model. The SIR model was initially derived to describe the spreading of a pathogen or disease in a population modeled as a contact network. However, it has since been applied to a variety of scenarios, from biological to social networks, as it captures the fundamental mechanisms involved in spreading processes, whilst being computationally and mathematically simple. Thus, we applied it here to model the propagation of ictal activity on a brain network from a set of seed nodes acting as the epileptogenic region (see Supplementary Figure S.1A, B). Each brain region (nodes in the network) can be in one of three states (S-I-R). Initially, all regions are susceptible (inactive, state S), except for a few active regions in the infected state (active state, I) that constitute the hypothesized epileptogenic zone (EZ). The active regions can propagate the activity to susceptible regions with probability β, but only if they are structurally connected. Thus, the probability that a certain region switches from susceptible to active at a given time step depends not only on β, but also on its number of active neighbors (neighbors are connected regions). Active regions can turn inactive again at each time step with probability γ, thereby becoming refractory such that they cannot be activated again (state R). Eventually, after many time steps the dynamics will reach a steady-state and all regions will be inactive (either in S or R states).

For a given network, the emerging behavior of the model depends only on the two control parameters β (probability of transition S → I) and γ (probability of transition I → R). Moreover, the dynamic behavior of the model is well known over a variety of network substrates, with a strong theoretical basis^40,42,47,48^. Whilst the SIR model is too simplistic to reproduce actual brain activity, it has been shown in the past that the SIS dynamics near the critical point is enough to reproduce relevant characteristics of functional brain networks^49^. In the case of seizure propagation, ictal activity can be regarded as an anomalous (infected) state that propagates over the system taking advantage of the existing connectivity. In fact, highly detailed models of ictal activity and seizure propagation usually introduce a slow variable to account for the propagation of the epileptic state throughout the brain. This is then coupled with one or more fast variables mimicking the activity of each region^27–29,32,50^. Considering the SIR model, we focus our analysis only on the propagation of ictal-like activity on the network, and analyze the effect of different virtual resections on propagation.

The overall progression of the seizure can be measured by the fraction of active nodes at each time step, I(t). I(t) follows a characteristic curve (a hyperbolic secant) when the spreading rate λ = β/γ is above the epidemic threshold λ_c_ of the network^40^. Initially, I(t) increases exponentially when the process is dominated by the propagation of ictal activity. Eventually, I(t) reaches a maximum and decreases as most nodes fall into the inactive state. The height of the maximum I_max_ characterizes the severity of the epidemic/seizure and, for a given network, it is correlated to the initial propagation speed^51^. We therefore characterized the severity of seizure propagation by the fraction of infected nodes at an early point of the propagation I(t_0_), with t*0* = 10. I(t_0_) captures how steep the slope of the propagation curve is at the beginning and indicates how fast the activity propagates.

Here, we ran the SIR model 10,000 times for 200 discrete time steps and averaged the number of active nodes at each time step over the runs. The parameters were chosen such that the activity propagated to the entire brain in most runs, i.e. on average 98% of the nodes were in the R state at the end of the run. We fixed γ = 0.03 and increased β in steps of 0.001 until this criterion was met. β varied between 0.027 and 0.038 for the analyzed patient cohort. The model was programmed and run in MATLAB (version R2012a and 2018b, The MathWorks Inc., Natick MA, USA).

### Surrogate for SIR model: Eigenvector centrality (EC) difference

We simulated the propagation of seizure activity as an epidemic process using the SIR model, and quantified the effect of a resection using the fraction of active nodes at time step 10. However, although this approach has a direct conceptual link with clinical practice, it was computationally too expensive in combination with the optimization method used to find optimal virtual resection strategies (see the section “Selecting connections: simulated annealing”). We have therefore used an alternative, faster approach to quantify the effect of a resection on seizure propagation based on a network metric, namely the reduction in centrality (after the virtual resection) of the hypothesized EZ nodes. In the following paragraph we explain the theoretical foundation for this choice.

The temporal evolution of I(t) in the SIR model depends both on the model control parameters—namely β and γ—and in the structure of the underlying network. For a given β and γ, I(t) is initially given (for very small t) by the degree (defined as the number of neighbors) of the seed nodes^40,52^, so that a larger degree results in a faster propagation. After a few steps, however, the degrees of the neighboring nodes, and of their neighbors and so on, also influence the dynamics as they become infected. Thus, not only the degree of the seed but also the topology of the network affects I(t). This global structure is reflected in another centrality measure: the Eigenvector centrality^53^ (EC). The EC of each node in the network indicates its participation in the dominant eigenmode of the adjacency matrix. In particular, for a diffusion process such as a random walk, the EC of a node indicates the probability that said node is occupied at a given time^51^. Although spreading and diffusion processes differ, they do share many similarities and the EC can be related to the speed of spreading of an epidemic starting at node I, as can be shown by the introduction of the pseudoinverse of the Laplacian^54^ (see also the SI, Sect. 2, for details). Therefore, in this study we used the EC as a surrogate for how ictal activity propagates on the network.

In order to explicitly validate the use of EC as a surrogate metric of seizure propagation on our patient-specific networks, we compared, for all patients, the EC of each node with the propagation at time t_0_ = 10 (i.e. I_t=10_) when the same node was use as the (single) seed for the SIR model (see Fig. 7). Moreover, we also compared the propagation using the whole resection area as the seed, with the EC of the seed, for all patients (Supplementary Figure S.2). The strength of the correlations was measured with Pearson’s correlation.

### Removing connections

#### Candidate connections for resection

The hypothesized EZ for each patient was based on the location of the resection area. The 3-month post-operative T1-MRI of each patient was linearly co-registered to a pre-operative T1-MRI using FSL FLIRT (version 5.0.10 with 12 parameter affine transformation). The AAL ROIs were overlaid on the co-registered post-operative scan. The ROIs that by visual assessment fell entirely or more than 50% within the resection area were labelled as hypothesized EZ nodes. To eliminate spread in the SIR model, all connections from infected to susceptible nodes should be removed^55^. Our aim was to keep the propagation of activity from the hypothesized EZ nodes to nodes outside the hypothesized EZ (hereafter called neighboring nodes) minimal from the start. Therefore, we considered all binarized DTI connections from hypothesized EZ nodes to neighboring nodes as candidate connections for removal in the virtual resections. Note that all these candidate connections had been removed during the actual surgery.

#### Measure of effect

**Figure 1:**
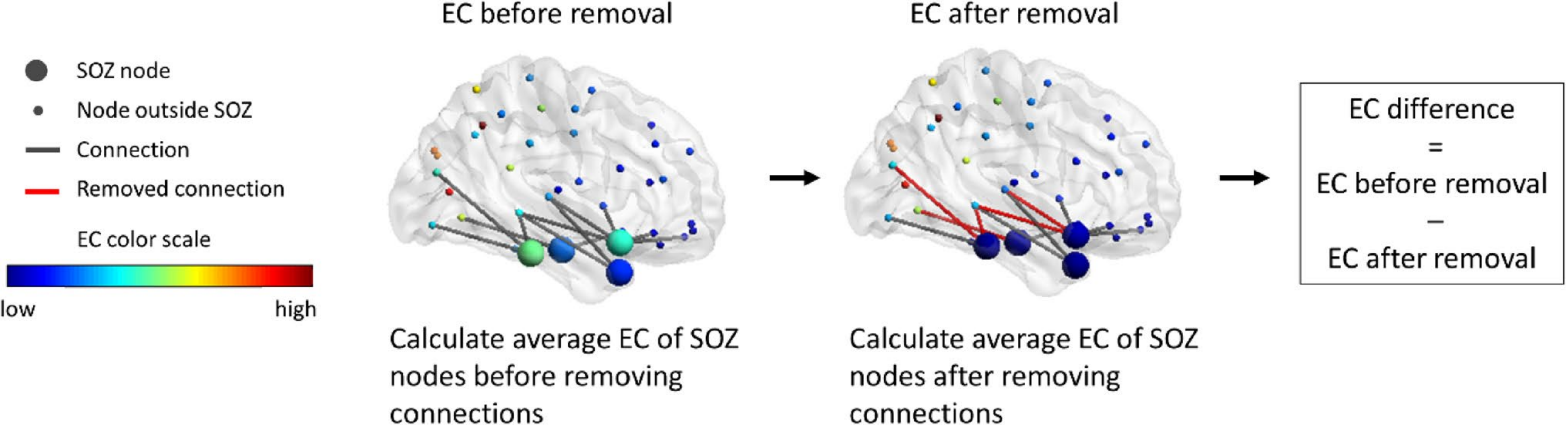
Illustration of the EC difference of the SOZ. The EC of each node is based on the full network (nodes inside and outside the SOZ). First, the EC is averaged over the nodes in the SOZ. After removing connections, the EC per node is re-calculated based on the resected network. Subsequently, the EC is averaged over the SOZ nodes again. The difference in EC of the SOZ nodes is the EC before removal minus the EC after removal. Parts of the figure were visualized with the BrainNet Viewer toolbox (Xia et al. 2013) (http://www.nitrc.org/projects/bnv/).

The choice of which connections to remove from the candidate connections was based on the effect of their removal, quantified here as the EC difference of the hypothesized EZ (Fig. 1). The EC difference is the difference in average EC of the hypothesized EZ nodes after removal of connections compared to the average EC before the removal. The EC difference, quantifying the reduction in centrality of the hypothesized EZ, was used as a surrogate measure of the reduction in speed with which a seizure propagates. In order to compare between different patients, we also used the normalized EC difference, defined as the fraction between the EC difference for a given resection and that for a total resection. Once an optimal virtual resection for a given size had been found using this surrogate approach (see below), the actual effect of the virtual resection was measured as the change in seizure propagation, given by I(t_0_). Similar as for the EC difference, we also defined the normalized change in seizure propagation, namely as the change in I(t_0_) after the resection was applied, divided by the change in I(t_0_) following a full resection. Note that, given that the seed remains in the network after the virtual resection, and is infected, I(t_0_) > 0 also for complete seed disconnection.

#### Selecting connections: simulated annealing

Generally, a large number of virtual resections are possible, meaning that many different combinations of connections can be removed. Testing each of these combinations constitutes an intractable combinatorial problem and we have therefore used simulated annealing^56^ as optimization method to find the optimal combination, i.e. the one resulting in the largest effect (the largest EC difference as a surrogate measure of largest reduction in the speed of seizure propagation). We used a MATLAB implementation of simulated annealing (J. Vandekerckhove, general simulated annealing algorithm, version 1.0.0.0., MATLAB Central File Exchange) to optimize for a large EC difference. As we aimed to maximize the EC difference, the algorithm minimized the negative value of the EC difference (object function = − EC difference). Simulated annealing optimized the combination of a fixed number of candidate connections (i.e. for a fixed resection size), after it was given an initial guess containing a random selection among all candidate connections. The algorithm considered neighboring states that were generated from the previous state, in our case by changing one of the currently selected connections. We used the default parameters of the implemented algorithm (initial temperature: 1, stopping temperature: 10^–8^, annealing schedule: f(T) = 0.8 T, maximum number of consecutive rejections: 1000, maximum number of tries within one temperature: 300, maximum number of successes within one temperature: 20).

**Figure 2:**
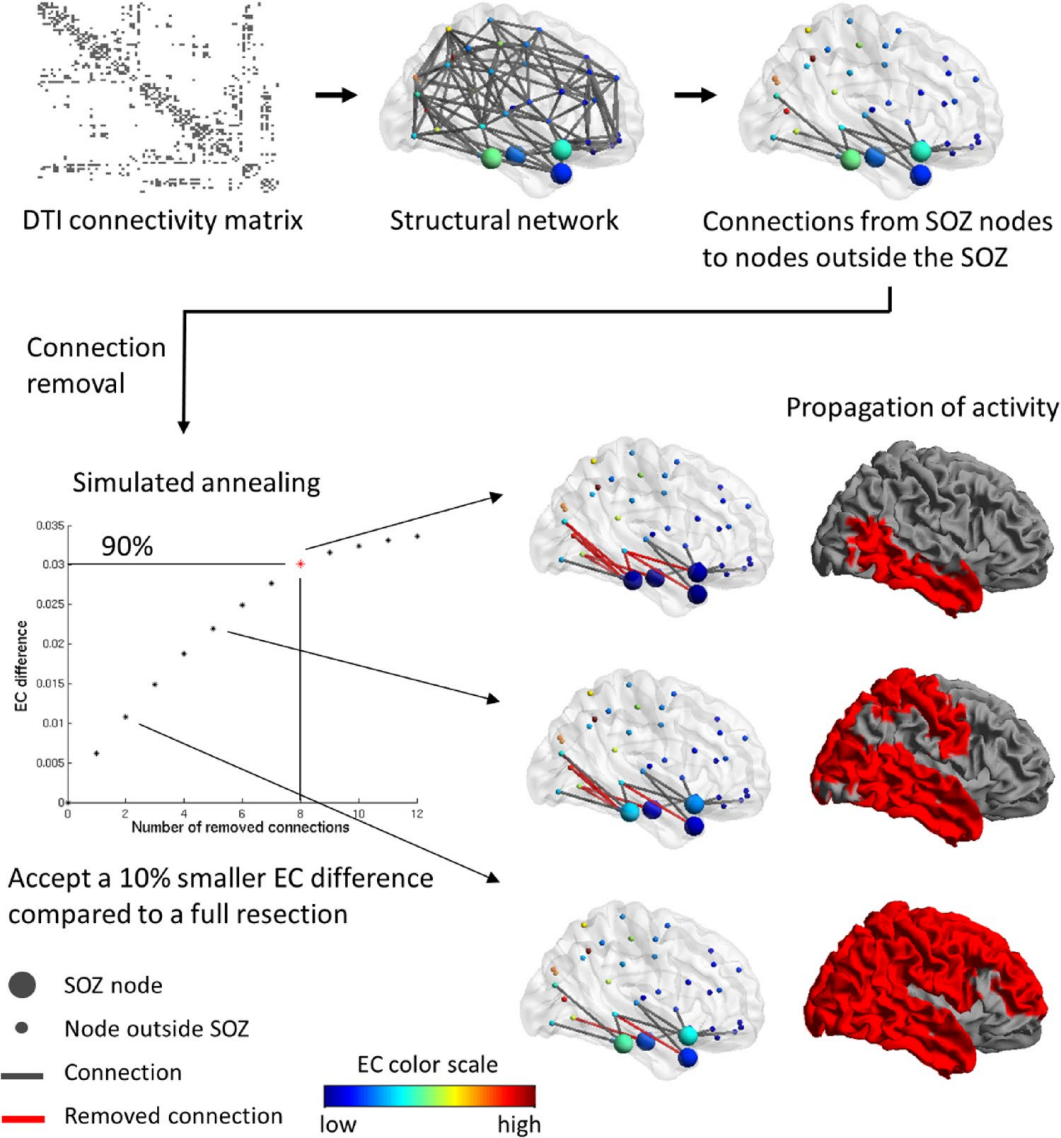
Workflow of selecting the connections to remove for each patient. The patient’s DTI connectivity matrix of white matter tract density between 92 ROIs is thresholded and binarized to a connectivity density of 11%. This connectivity matrix is the basis for the structural network. Subsequently, the connections from SOZ nodes to nodes outside the SOZ are identified—they were all severed in the surgery. Those connections are candidates for the virtual resection in our model. For each number of removed connections, the optimal choice of connections is found using simulated annealing. Simulated annealing optimizes for those connections that upon removal result in the largest EC difference of the SOZ. After the maximal achievable EC difference has been found for each number of removed connections, the number of connections yielding 90% of the EC difference compared to the maximum (when removing all connections) is found and removed. Because removing the first few connections has a larger effect than removing further connections, a relatively large number of connections can be spared yet still achieving an almost maximal effect. The rationale behind the method is illustrated with the propagation of activity (red brain regions indicate active regions at the same time after seizure onset): removing the connections that decrease the EC of the SOZ results in a decrease in speed with which activity propagates. Parts of the figure were visualized with the BrainNet Viewer toolbox (Xia et al. 2013) (http://www.nitrc.org/projects/bnv/).

Simulated annealing was repeated for different resection sizes, hence an optimal EC difference was obtained for each resection size. The largest global EC difference is achieved when removing all candidate connections, which equals the EC of the hypothesized EZ in the un-altered network. This trivial solution with an effect of 100% was used as the reference point. However, the relation between the resection size and optimal EC difference was non-linear: the first removed connections contributed more to the effect than the last removed connections. In fact, for larger resection sizes the EC difference grew sub-linearly, until increasing the size led to only a small subsequent increase in the EC difference. This means that we can accept for instance a 10% decrease in effect, while sparing substantially more than 10% of the connections. Here, we chose a resection with a slightly smaller effect (10%), but where several connections could be spared (Fig. 2). We defined the optimal resection (with a given number of connections, referred to here as the optimal size) as the one that achieved 90% of the effect of a full resection. The actual threshold is thus arbitrary, chosen here to take advantage of the non-linear dependence of the EC difference on the resection size and to maintain most (90%) of the effect of the full resection. Small changes in this threshold also would lead to small quantitative changes in the results, but would not affect the main findings. To summarize, the goal was to reduce seizure propagation by removing connections and at the same time sparing the brain as much as possible.

### Comparison to other selection methods that are based on network measures or random selection

So far, the effect that removal of a connection (i, j)—where i is a node in the hypothesized EZ and j a neighboring node that does not belong to the EZ—had on the EC of the hypothesized EZ (in combination with the removal of other connections) determined whether that connection was included in the optimal resection. Other criteria could be used to determine the importance of a connection in the structural network based on different centrality measures:

1. Betweenness centrality of the edge (i, j) (edge BC), defined as the number of shortest paths in the network that go through the edge (i, j)^57,58^,
2. Eigenvector centrality (EC) of the neighboring node j^53^,
3. Degree (defined as the number of neighbors) of the node j^37^, and
4. Betweenness centrality (BC)^59^, of the neighboring node j.

The brain connectivity toolbox in MATLAB (version 2017-15-01)^60^ was used for calculating edge BC, EC and BC. We determined the effect of a resection with the optimal size (the same size as found by the simulated annealing algorithm), but choosing the connections based on these network measures. For comparison, the effects of resections with the same number of randomly selected connections among the candidate connections were also computed (average and standard deviation for 100 random selections). For the comparison, we used both the actual effect of a resection, as given by I(t_0_), and the surrogate metric given by EC difference of the resection.

### Statistics

The network characteristics of the connections from the hypothesized EZ that were spared were compared to those that were removed using an unpaired Student’s *t* test. We compared the (i) edge BC, and the (ii) EC, (iii) degree, and (iv) BC of the node outside the hypothesized EZ to which a removed/spared edge was connected. The *p*-values were corrected for multiple comparisons using the false-discovery rate (FDR)^61^.

We also compared the efficacy of the resections found by different resection strategies, as given by both the EC difference and the propagation I(t_0_), using a paired Student’s *t* test (significance level: 0.05). Finally, we used an unpaired Student’s *t *test to compare the initial propagation I(t_0_) (before any resections), the EC of the seed (equal to the EC difference of a full resection) and the propagation I(t_0_) after the optimal resection of the seizure-free patients to those for the patients who were not seizure-free. The two surgical outcome groups were also compared for size (unpaired Student’s *t *test) and location (Chi-square test) of the hypothesized EZ, as indicated by the number of removed nodes and the indices (in the AAL atlas) of these nodes, respectively.

## Results

The structural brain networks of 19 patients with temporal (*n* = 15) and extratemporal (*n* = 4 frontal) epilepsy were analyzed. After surgery, 14 patients were classified as Engel class 1 and were labelled as SF, whereas 5 patients were classified as Engel class 2–4 and labelled as NSF. First, the analysis was applied to all patients individually, followed by a comparison of the two surgical outcome groups.

**Figure 3:**
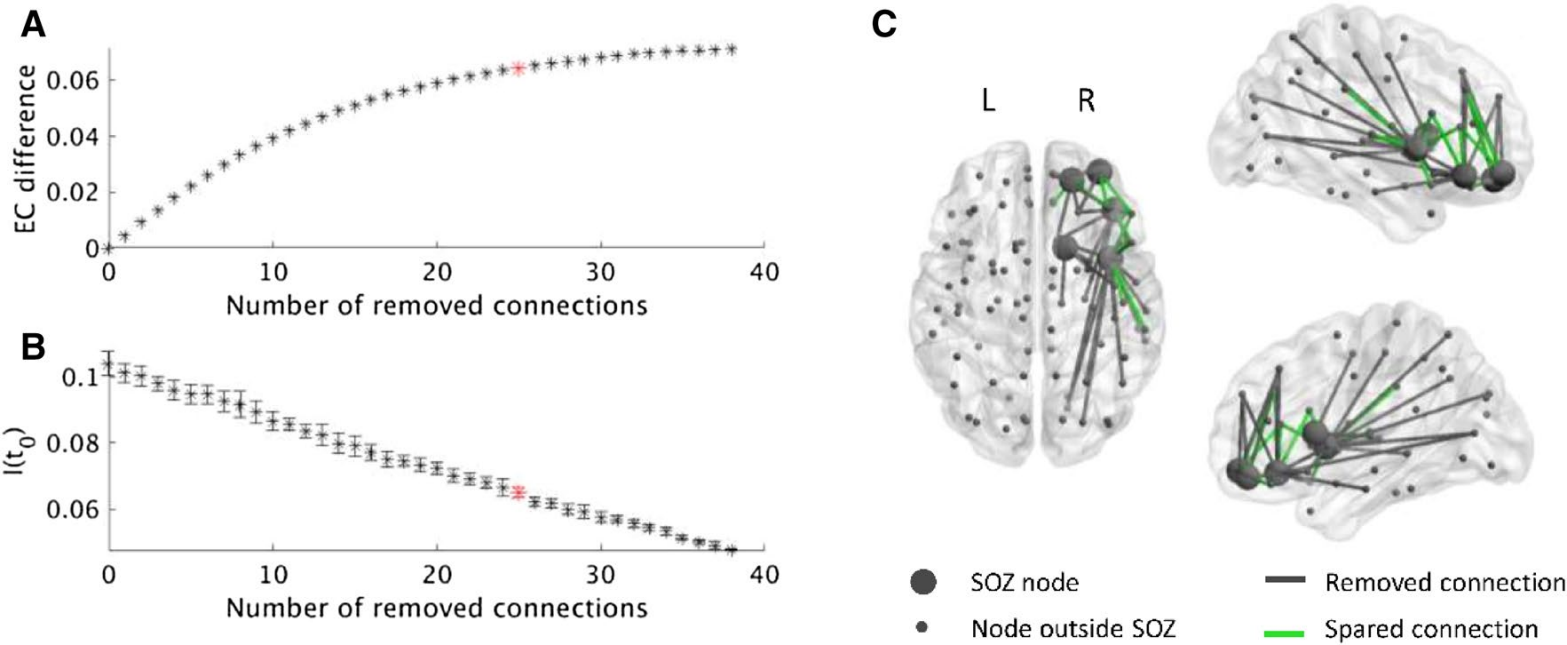
Example of selecting the connections to remove for patient 15. (**A**) The selection was found separately for each number of connections using simulated annealing and the EC difference. After the removal of some connections from the SOZ to the rest of the brain, the EC difference of the SOZ increased. Removing all connections as in the surgery resulted in the highest EC difference. However, a 10% smaller decrease in EC was achieved by removing only 25 of the 38 connections, thereby sparing 13 connections (red star). (**B**) We calculated the actual effect of each resection on the seizure propagation model by measuring I(t_0_) after the resection took place. I(t_0_) decreases in a roughly linear manner with the number of removed connections. The red star indicates the resection corresponding to a 90% decrease in EC difference. Errorbars indicate the standard deviation among 10 iterations of the SIR model averaged over 1000 realizations. (**C**) The connections from the SOZ to the brain regions outside the SOZ are displayed, including the 13 spared connections (green). Parts of the figure were visualized with the BrainNet Viewer toolbox (Xia et al. 2013) (http://www.nitrc.org/projects/bnv/).

For each patient, the virtual resection was optimized by simulated annealing, for each resection size. An example of the analysis for one of the patients is shown in Fig. 3. The optimal EC difference increased non-linearly as more connections were removed (Fig. 3A); this was the case for all patients. Seizure propagation (given by I(t_0_)) decreased with the resection size, in an approximately linear manner (Fig. 3B). The non-linearity in EC difference meant that the first removed connections had a greater contribution to the total effect than the last removed connections. Trivially, the largest EC difference was achieved when all connections were removed, i.e. corresponding to the resection that was performed during the actual surgery. Accepting a 10% smaller EC difference compared to the maximal EC difference spared 13 of the 38 connections (34.21%, which is substantially more than 10%) in this example. This meant that 90% of the effect was obtained by removing only 25 (65.79%) of the candidate connections. The spared and removed connections are displayed in Fig. 3C. To conclude, the virtual resection was one-third smaller in this patient than the actual surgery, whilst achieving almost the same (90%) effect in terms of reducing the EC of the seed.

### Analysis of optimal virtual resections: EC

**Figure 4:**
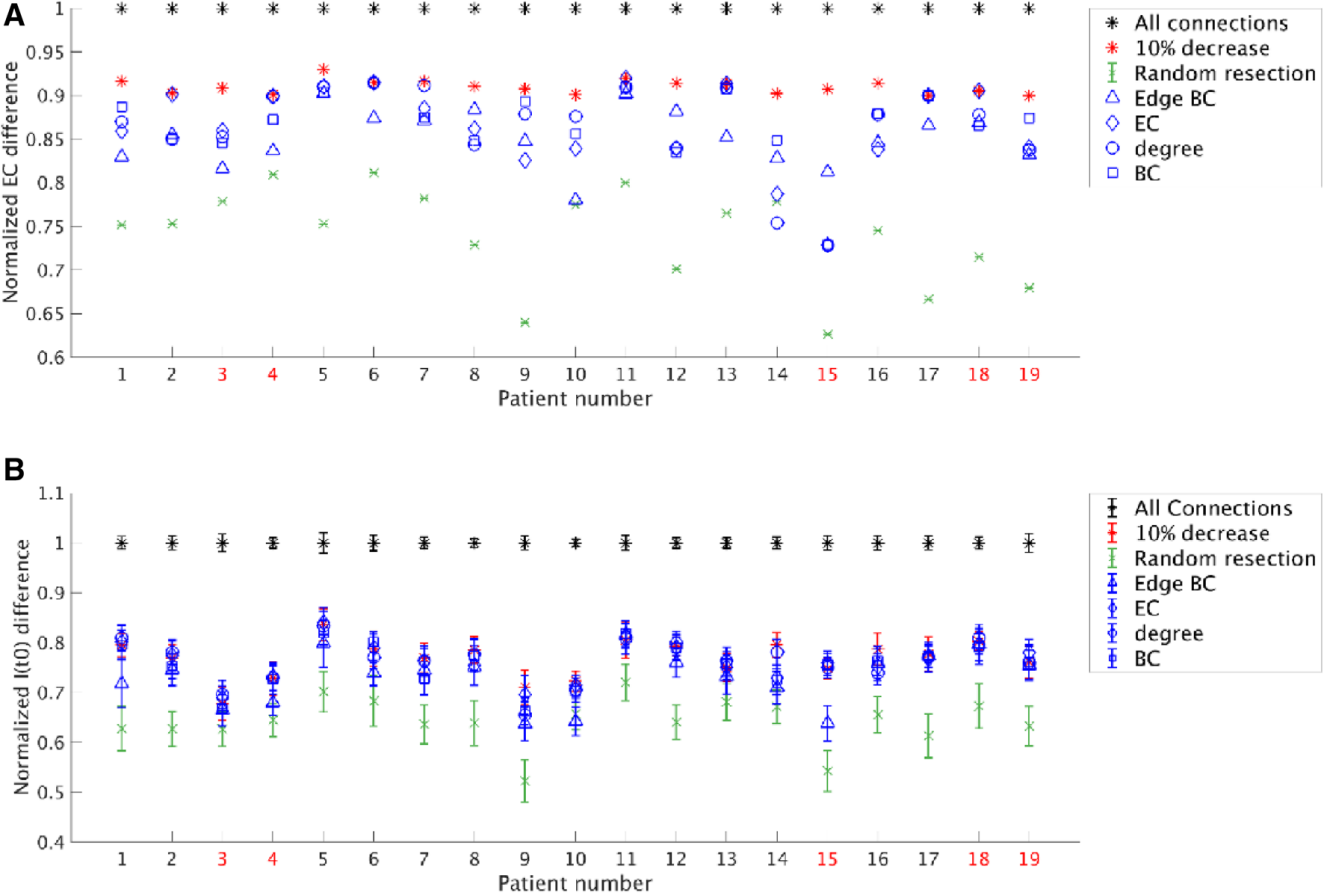
Comparison of various resection strategies for all patients as measured by the normalized EC difference and the normalized difference in I(t_0_), respectively in panels (**A**) and (**B**). (**A**) The normalized EC difference for a resection is defined as the ratio between the EC difference for the optimal resection and the EC difference for a full resection. Removing all connections resulted in the largest EC difference (100%, black stars). A 10% decrease in the EC difference was accepted (red stars), using simulated annealing to optimize which connections to remove, and thereby sparing connections. This method performed better than the average of 100 random resections (green cross with standard deviation), using the same number of removed connections chosen randomly from the candidate connections. For comparison, the same number of removed connections were also chosen using network measures: edge BC (blue triangles), EC (blue diamonds), degree (blue circles), and BC (blue squares). The three latter measures were based on the property of the connected node outside the SOZ. (**B**) The normalized I(t_0_) difference for a resection is defined as the difference between I(t_0_) before and after the resection, normalized by this difference for a full resection. Similarly to the EC difference, removing all connections (black stars) resulted in 100% decrease of I(t_0_). The optimal resection given by the surrogate model (red stars) performed marginally better than the resections based on network metrics (blue markers) for some patients. Random resections (green markers) performed the worst. All values correspond to the same resections used in panel **A**. The error bars were calculated as in Fig. 3. The not seizure-free patients are marked in red in both panels.

The optimization results for all patients are shown in Fig. 4A. Our optimization approach of sparing connections whilst achieving a 90% effect (average: 90%, standard deviation: 0.8%) was compared to other resection strategies based on network characteristics, or random resections. On average, 27.49% (standard deviation: 4.65%) of the connections were spared when allowing for a 10% reduction in effect. The optimization strategy performed better (obtaining a larger reduction in EC) than a random resection of the same size in all patients which, on average, led to a 36% (standard deviation: 6%) decrease in effect. The difference (90% effect-random = 0.170) was significant (*t*(18) = 13.80, *p* = 5e–11). However, the effect varied between patients: in some patients optimizing the resection strategy yielded much better results than in others.

The removal of connections on the basis of their, or their connected neighboring nodes’, network characteristics was also more effective on average than a random resection [mean decrease in effect (and standard deviation): edge BC: 0.15 ± 0.30, EC: 0.14 ± 0.05, degree: 0.13 ± 0.13, BC: 0.13 ± 0.04], and the difference was significant: edge BC-Random = 0.112, *t*(18) = 8.02, *p* = 2e–7; EC-Random = 0.125, *t*(18) = 10.65, *p* = 3e–9; degree-Random = 0.125, *t*(18) = 9.64, *p* = 1.6e–8; BC-Random = 0.128, *t*(18) = 10.60, *p* = 4e–9. Moreover, the removal based on the network metrics performed better than the random removal for most individual patients (edge BC: 18/19 patients; EC: 17/19 patients; degree: 17/19 patients; BC: 18/19 patients).

In all patients, our approach performed better than or equally to optimization based on the network characteristics of the connections or their connected neighboring nodes. On average, the optimization approach performed significantly better than all network metrics (optimal-edge BC = 0.058, *t*(18) = 9.16, *p* = 3e–8; optimal-EC = 0.045, *t*(18) = 4.11, *p* = 7e–4; optimal-degree = 0.045, *t*(18) = 4.00, *p* = 8e–4; optimal-BC = 0.042, *t*(18) = 4.56, *p* = 2e–4). However, there were differences among the network measures in the effect that could be achieved. In one patient (patient 11), all of the network measures performed equally to our optimization approach. In all other patients, the largest effects among the network measures were achieved when the resection strategy was based on the EC of neighboring nodes to which outgoing edges of the hypothesized EZ connected (note: not to be confused with the EC difference of the hypothesized EZ), followed by BC, degree and edge BCs. The resection strategies based on network measures achieved the same effect as our optimized strategy in 8 patients. This shows that some connections, or the neighboring nodes to which they are connected, are more important than others in achieving a large effect. The most important connections are those that connect the hypothesized EZ to a neighboring node with high EC. There were no significant differences in the average performance of the edge BC, EC, degree and BC based resections.

### Analysis of optimal virtual resections: I(t_0_)

We repeated the comparison with random resections and resections based on network metrics for the actual effect of the resection (measured as the decrease in I(t_0_)). The results for all patients and resection strategies are shown in Fig. 4B. On average, the optimal resection achieved a 77% ± 4% decrease in propagation, random resections achieved a 64% ± 5% decrease, whereas the network metric based resections achieved a 73% ± 5%, 76% ± 4%, 76% ± 4% and 76% ± 5% decrease, respectively for the edge BC, EC, degree and node BC.

As in the previous analysis, we found that the optimal resection performed better than a random resection for all patients, and significantly better on average (optimal-random = 0.127, *t*(18) = 13.25, *p* = 1.0e–10). It also performed better on average than the network metrics based resections, although the difference was only significant for the edge BC and nodal BC metrics (optimal-edge BC = 0.039, *t*(18) = 5.12, *p* = 7e–5; optimal-EC = 0.006, *t*(18) = 1.36, *p* = 0.19; optimal-degree = 0.005, *t*(18) = 1.23, *p* = 0.2; optimal-nodal BC = 0.012, *t*(18) = 2.53, *p* = 0.021). Moreover, resections based on edge BC performed on average significantly worse than resections based on other network metrics (EC-edge BC = 0.033, *t*(18) = 4.20, *p* = 5e–4; degree-edge BC = 0.033, *t*(18) = 4.38, *p* = 4e–4; nodal BC-edge BC = 0.026, *t*(18) = 3.45, *p* = 3e–3). Overall, resections based on the nodal degree or the EC had a larger effect on propagation than resections based on BC, as expected from theory: the node’s degree and EC strongly influence SIR dynamics (Barrat et al., 2009; Pastor-Satorras et al., 2015) (although the differences among the performance of the resections based on the EC, degree and BC of the neighboring node were not significant).

The resections based on the network metrics also performed better than random resections for all patients, and significantly better on average (edge BC-Random = 0.088, *t*(18) = 8.98, *p* = 5e–8; EC-Random = 0.121, *t*(18) = 11.87, *p* = 6e–10; degree-Random = 0.121, *t*(18) = 12.85, *p* = 1.7e–10; BC-Random = 0.114, *t*(18) = 11.39, *p* = 1.2e–9).

### Characteristics of removed connections

**Figure 5:**
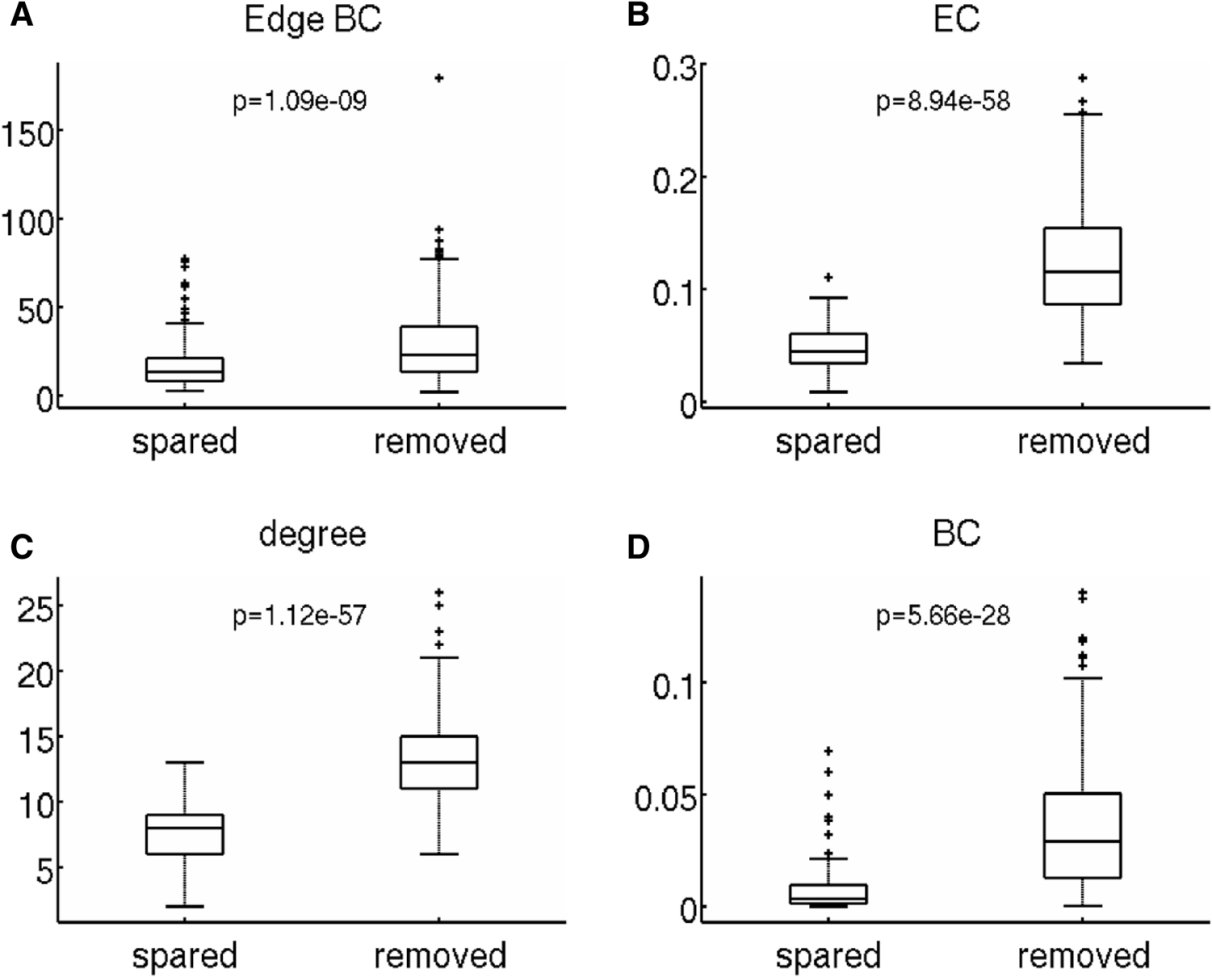
Do the network characteristics of the spared and removed connections differ? A 10% decrease in EC difference spared several connections (compared to a full resection). The network characteristics of the connections that were spared were compared to those that were removed. The network characteristics were edge BC of the connection (**A**), as well as EC (**B**), degree (**C**), and BC (**D**) of the node outside the SOZ that was connected to a SOZ node with the connection in question. Connections to hub nodes were removed significantly more often than they were spared. The *p*-values were corrected for multiple comparisons using FDR.

These results lead to the question: do the network measures differ between the spared and removed connections? To this end, we compared the network characteristics of the spared versus the removed connections (Fig. 5). All network measures were significantly different between the two groups of connections: edge BC (*t*(546) = − 6.20, 95% CI = (− 14.68, − 7.62), *p* = 1.09e-9), EC of the neighboring node (*t*(546) = − 18.24, 95% CI= (− 0.09, − 0.07), *p* = 8.94e-58), degree of the neighboring node (*t*(546) = − 18.15, 95% CI= (− 6.57, − 5.29), *p* = 1.12e-57), and BC of the neighboring node (*t*(546) = − 11.63, 95% CI= (− 0.03, − 0.02), *p* = 5.66e-28 ). Hence, central connections and connections from the hypothesized EZ to hubs were removed significantly more often than they were spared. This makes sense intuitively: removing hub nodes or central connections limits the propagation of activity across the network. Thus, the network measures indicated that connections to neighboring hubs, or central connections, should be removed.

### Surgery outcome

**Figure 6:**
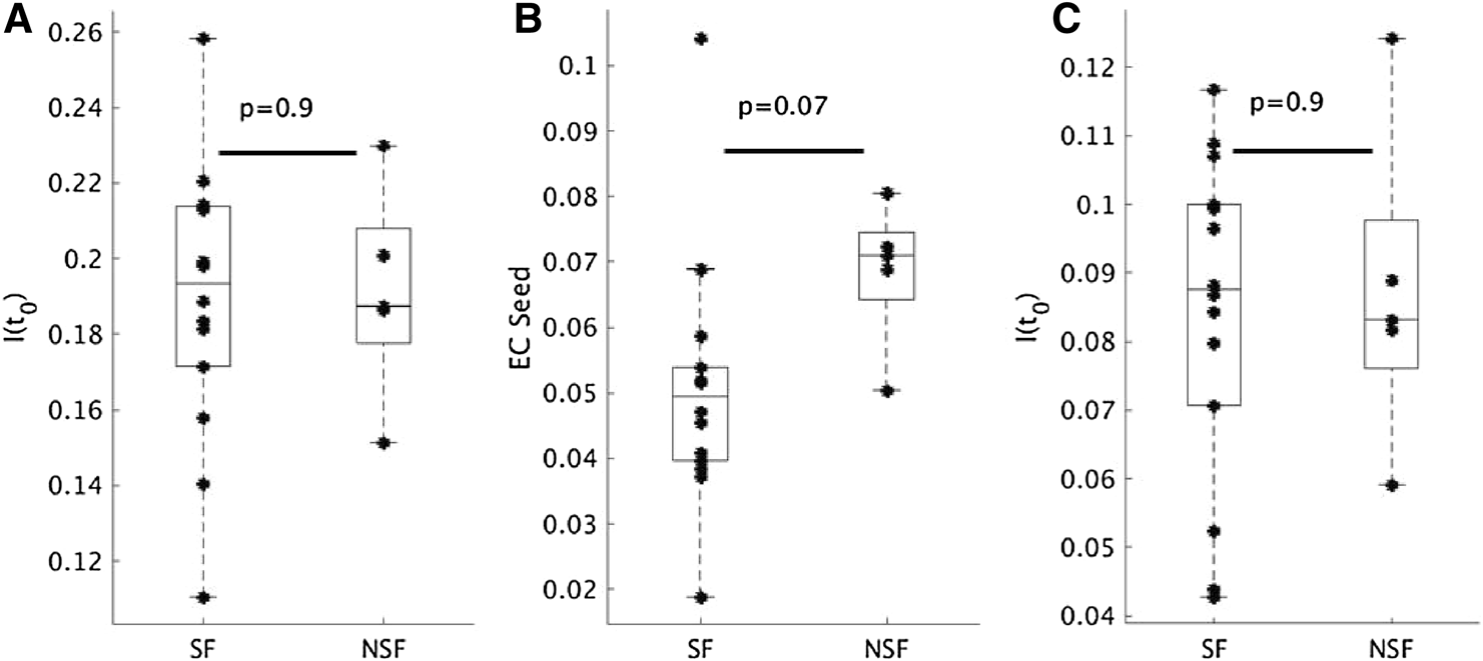
Seizure-free (SF) compared to not seizure-free (NSF) patients. (**A**) The seizure propagation as measured by I(t_0_) before the virtual resection was not different between the SF (*n* = 14) and NSF (*n* = 5) patient groups. (**B**) The EC of the seed (which is equal to EC difference for a full resection) was marginally larger for the NSF group than for the SF group, although the difference was not significant (*t*(17) = 1.95, *p* = 0.07). (**C**) The seizure propagation after the optimal resection that led to a 90% EC difference, measured by I(t_0_) after the resection, did not differ between the groups. The groups did not differ in the size or location of the SOZ.

We compared the surgical outcome groups to differentiate between the success of the optimized resection plans. The previous analysis (Fig. 4) did not indicate any obvious differences between SF and NSF with regards to the performance of different resection strategies. In Fig. 6 we compared the two groups with respect to seizure propagation (as measured by I(t_0_), panels a and c) and EC of the hypothesized EZ (panel b). Initially (before any resections), there was no difference in propagation between the groups (Fig. 6a), but we found a marginal, non-significant difference (*p* = 0.07) in the EC of the hypothesized EZ (which is equal to the EC difference, Fig. 6b), with the NSF group having a larger EC difference than the SF group. Consequently, the absolute decrease in EC difference for a total resection and for the optimal resection was also (not significantly) larger for the NSF group compared to the SF group (data not shown), since these metrics equal respectively 100% and 90% of the EC of the seed itself. We did not find a significant difference between the two groups in propagation I(t_0_) for the optimal resection either (Fig. 6c). The two groups did not differ in the size (*t*(17) = 0.26, 95% CI = (− 2.09, 2.69), *p* = 0.78 or location (temporal versus extratemporal: χ^2^(1) = 0.0045, *p* = 0.95) of the hypothesized EZ.

### Effect of the network backbone

Using the averaged structural matrix, the number of candidate connections was 26.89 ± 6.81, compared to 28.84 ± 7.79 using the individual matrices (see Supplementary Information Sect. 3, Supplementary Figure S.3 and Supplementary Table S.1). There were no significant differences between using the average or individual matrices in the fraction of connections that had to be removed to achieve a 90% effect (paired *t*-test: *t*(18) = − 2.09, *p* = 0.51) or in the achieved EC difference at 90% (paired *t*-test: *t*(18) = − 1.38, *p* = 0.19).

Using the weighted structural matrix without thresholding, the number of candidate connections increased from an average of 28.84 ± 7.79 in the thresholded and binarized network to an average of 312.68 ± 109.59 in the weighted network without threshold, as all connections were included (see Supplementary Information Section 4, Supplementary Figure S.4 and Supplementary Table S.2). The EC difference at 90% did not differ significantly between the two analyses (paired *t*-test: *t*(18) = 1.38, *p* = 0.18). However, a significantly smaller fraction of connections had to be removed for the weighted network (0.04 ± 0.01) compared to the binary network (0.72 ± 0.05) (paired *t*-test: *t*(18) = 60.55, *p* = 2.94e-22). This result is not surprising, given that in a weighted network the strongest candidate connections have an unproportionally high influence on the EC and, consequentially, the removal of the few strongest ones suffices to decrease the EC to 90% of the full effect. To alleviate this unproportional contribution to the EC, we took the logarithm of the weights and repeated the analysis. For the weighted log networks (see Supplementary Information Section 4, Supplementary Figure S.5 and Supplementary Table S.3), there was no significant difference in the fraction of removed connections (0.74 ± 0.07) compared to the binary networks (0.72 ± 0.05) (paired *t*-test: *t*(18) = − 1.08, *p* = 0.30). Additionally, the weighted resection was mostly included in the binary resection (average: 83%).

### Validation of surrogate model

**Figure 7:**
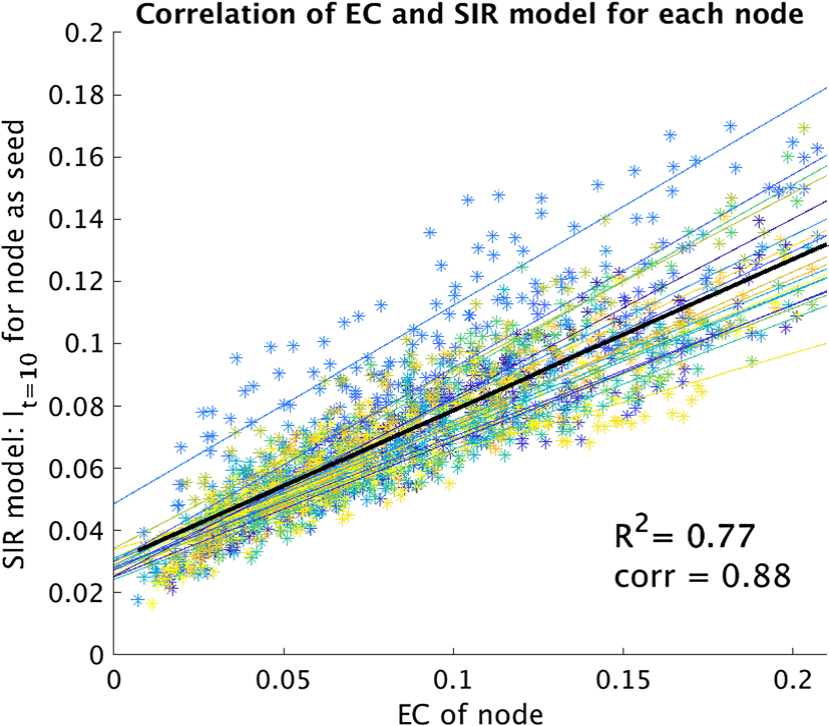
Correlation of the nodal EC with the SIR model for all patients. The EC was calculated for each of the 92 nodes in the structural network. For the SIR model, each node was activated separately at the start (only one seed) and the number of active nodes at time 10 (I_t=10_) was averaged over 10,000 runs. The correlation was measured independently for each patient (thin coloured lines); on average the Pearson’s correlation coefficient was 0.95 ± 0.02. When considering all data points together, the correlation decreased to 0.88 but was still strong.

For the validation of the EC of the hypothesized EZ as a surrogate for seizure propagation, we compared, for all patients, the EC of each node with the initial speed of propagation (I_t=10_) in the SIR model (Fig. 7), which revealed a strong correlation with an average of 0.95 ± 0.02. Moreover, we also compared in the Supplementary Information (Supplementary Figure S.2) the propagation I_t=10_ when using the whole resection area as the seed with the EC of this seed, rescaled by the seed size, for all patients. We found a strong correlation of 0.78 despite the fact that each data-point corresponded to a different patient-network set. It is therefore valid to use the EC difference of the hypothesized EZ as a proxy for the speed of seizure propagation in the SIR model.

Taken together, the effect of our approach varied among patients and certain connections contributed more than others. Removal of connections to hub nodes or central connections resulted in a larger effect, as this probably limits the propagation of activity. NSF patients showed a larger decrease in EC than SF patients after connection removal (although the difference was not significant), which was due to a large EC of the hypothesized EZ before the resection.

## Discussion

### Summary

We presented a computational model based on structural brain networks of individual patients and evaluated the effect of virtual resections on seizure propagation in a preliminary study. Together with the SIR model, we developed a surrogate, namely the EC difference, which correlated strongly with the SIR model results and was computationally feasible. Additionally, we solved the computational problem of not being able to test all possible combinations of connections by using simulated annealing, which selected the optimal combination of connections. We found that, when allowing for a 10% decrease in effect compared to removing all connections as in the actual surgery, substantially more than 10% of the connections could be spared. This means that we were able to make smaller resections with almost the same effect as the actual surgery in our patient cohort. This approach performed better than a randomly selected removal of the same number of connections, and better or equal to removal based on network measures. For the network measures, the best performance was achieved by a selection based on the EC of the neighboring node outside the hypothesized EZ to which a removed connection was connected. Importantly, the effect of our approach varied per patient. Some connections contributed more to the effect than others: the connections to neighboring hub nodes were preferably removed. NSF patients had a tendency to a larger EC of the hypothesized EZ before the virtual resection (although the difference was not significant, *p* = 0.07), which could not be explained by differences in size or location of the hypothesized EZ. This may suggest that patients with a stronger pathological hub in the SOZ may need a more extensive resection or disconnection to become seizure-free.

### Smaller resection area

We found that with a slightly smaller effect than the actual surgery many connections could be spared. Our reference was the removal of all connections that were cut during the actual surgery, namely the connections from the hypothesized EZ to the rest of the brain. A roughly similar reference was taken by Olmi and colleagues, who in their model also removed connections selected from those cut during the actual surgery^27^ and started modelled seizures in the hypothesized EZ. Instead of measuring the effect of removing connections (as in our approach), Olmi et al*.*^27^ aimed for the same effect that the actual surgery had in the model, namely no seizure propagation. They found that it was possible to achieve the same effect as the actual surgery with removing far fewer connections. Moreover, they also found that the model-based virtual resections outperformed those based on random resections or on the network metrics alone, as in our study. Olmi and colleagues also found that some network characteristics of the seed regions predicted better than others the number of removed connections in the optimal resection. In particular, they found the node efficiency, clustering coefficient and betweenness centrality to be stronger predictors of the number of removed connections than node strength, degree or closeness centrality. This relates to our finding that the network characteristics of the removed and spared connections are different, and similarly we also found a larger difference for the node betweenness centrality than for the degree. Thus, although not enough to define the optimal resection, network metrics can guide the first assessment of a proposed virtual resection. The two analyses are fundamentally different, however, since the former refers to the characteristics of the seed nodes, and the later to those of the removed connections, for a given seed and resection. Finally, as these authors suggest in their concluding remarks, their approach is more likely to be palliative than curative in terms of achieving true and lasting seizure freedom, whereas we provide actual follow-up in our patient population (SF and NSF groups).

Our study is in line with previous studies suggesting that individualized tailoring of resections can be performed using personalized computer models^22–24,29,32,33^. A tailored, often smaller resection area implies that less brain tissue is removed, and/or that surgery is performed using appropriate and patient-specific disconnection strategies^62^. Moreover, selecting individual connections, instead of removing whole brain areas, would allow for even smaller and less invasive resections. Patients with complex extratemporal resections and patients with an overlap of the planned resection with eloquent cortex stand to gain the most. Smaller resections may result in fewer side-effects and cognitive complaints after surgery^17^, thereby improving the quality of life of the patient. The current study is only a proof-of-principle of the use of spreading models and tailored resections to aid epilepsy surgery, and whereas current surgical techniques are based on removal of whole areas, advances in surgical methods like the gamma knife^62^ or real-time visualizations of anatomical connections during surgery^63^ allow for more selective surgery.

### Network measures

We found that all network measures identified the optimal selection of connections to be removed moderately well, even though they did not achieve the same effect as our model. Specifically, selecting connections for removal based on the hub measures (EC, BD or degree) of the neighboring node performed better than edge BC. Previous studies also found that resections based directly on network metrics performed relatively well^24,27,29^. Based on these results, it might not be necessary to use the dynamical model for selecting the nodes or connections for removal, but only for evaluating the effect of the removal. This approach has been used in some studies that selected the nodes to be removed based on network measures—such as the out-degree or a modularity analysis—directly^22,31^. These results show that network topology is an important indicator of the dynamics that are generated by the models. Could network measures even replace the use of computationally expensive models? Models add dynamics to the network, and therefore mimic the modelled event (propagation of seizures) closer than static network measures do. On the other hand, these dynamics can seemingly be approximated by network measures, which are much faster to calculate and do not need parameter estimation, especially at the earliest stages of the propagation^40, 64^. For example, the EC represents the probability that a random walker is at a randomly chosen node at any given time. In our study, hub measures were chosen because of their putative relation to spreading processes, which might explain why they were effective in some patients. However, the network measures related to the connections or neighboring nodes did not always perform as well as our model and could not replace the model in our setup. Nonetheless, if the network measures can perform as well as the models in all patients, they are preferable due to the straightforward and fast calculation. However, more refined work on this topic is needed to show which network measure or combination of network measures are best suited.

### Removed connections link the hypothesized EZ to hubs

Previous studies have indicated that the EZ might act as a hub, from which seizure activity propagates to the rest of the brain^9,65^. Yang et al.^31^ took this idea as the basis for their computer model by using a hub measure (out-degree) as an indicator of the EZ^31^. Another model study showed that the epileptogenic nodes in the model are hubs^23^. Our study, however, suggests that, possibly more important than removing the hub in the EZ, the hub connecting the EZ to the rest of the brain should be removed^66^. This result is not surprising, as the propagation pathways depend on the underlying network structure^67^ and hubs facilitate early propagation^68^. If the hubs are no longer connected to the EZ, propagation of seizure activity to the rest of the brain is hampered. Currently, it is unclear whether the EZ itself constitutes a hub or is connected to a hub^65,69,70^. In comparison to a hub-less EZ, many more connections need to be severed to stop seizure propagation in the case that the EZ is a hub itself^27^. This suggests that localizing the (pathological) hub might be more effective and result in a smaller resection area than localizing the SOZ. Two other model studies give further evidence that hubs should be removed during surgery^24,25^. Whether the hubs are inside or outside the EZ, these results suggest that hubs should be the target for surgery, rather than the SOZ. Generally, alternative resection strategies that do not encompass the SOZ might be successful^71^, and would be useful in cases where the SOZ is located in eloquent cortex^10^.

### Methodological considerations

The main analysis results using the individual structural matrices did not differ significantly from the results obtained using an averaged structural matrix. This might be because of only small variation in the DTI’s of the patients (the thresholding and binarization even further decrease variation), and because of the small patient number (each patient’s individual matrix constitutes 1/19 of the average matrix). These results mean that for this study we could potentially have used the average matrix, but we chose to stay with the individual matrices, as we hope to extend this study to more patients and future refinement of the analysis might expose advantages of using the individual matrices. Our goal was a model tailored to the individual patient and hence we stick with individual data where it is available, even though the results in this analysis did not differ from results with the average matrix.

The main analysis results using the binarized and thresholded structural matrices did not differ significantly from the results obtained using a structural matrix with the logarithm of the weights. This similar fraction of removed connections supports our choice for a threshold, even though finding the optimal threshold is a recognized issue in brain network science^72^.

In clinical practice, the ultimate goal is seizure freedom, though a significant reduction in seizures results in a considerable increase in quality of life of the patient. For our surrogate measure (EC difference), we chose to achieve maximally 90% of the effect of a full resection, as achieving 100% is trivial in our approach—the EC of an entirely disconnected SOZ would always be zero. The choice to find a balance between a large effect and a minimal resection means that seizure reduction is achieved rather than seizure freedom. The choice of the threshold value (90%) took advantage of the non-linear dependence of the EC difference on the size of the virtual resection, such that for larger resections it grew sub-linearly and increasing the number of resected edges only led to a small subsequent increase of the EC difference. The actual value of the threshold is arbitrary and other values could have been used as long as they fall within this regime. Here, 90% was selected as an standard value that fell within the saturating regime and only led to a small (10% or less) decrease in efficacy of the resection. Small variations in the threshold could weakly affect our quantitative results, but our main findings—namely the existence of smaller resections with only a small decrease in efficacy—do not depend on this actual value, and would thus hold. This model is a first step towards a more refined model that would have to be tested in a clinical population.

### Strengths

The main advantage of our model study is its simplicity. There are only few parameters that need to be estimated for the SIR model, and none for the proposed surrogate approach that minimized the EC of the hypothesized EZ. The model might show to give reproducible results in future studies, which is an important requirement for a computer model to be implemented in epilepsy surgery^18^. So far, we have achieved virtual resections that were more effective than random resections with a very simple model and even without considering resections in different locations away from the hypothesized EZ.

Our model used structural instead of functional networks. In this way the structural pathways (‘roads’) are captured on which seizures propagate. However, these roads may be ‘open’ or ‘closed’ for the activity propagation^25^. We assumed that the model simulates functional activity that may or may not propagate, so that this process imitates the open or closed roads. According to this line of reasoning, simulating functional activity on a network of functional connections would be irrational^73^. We have therefore captured a more realistic setting: functional activity propagating on a structural backbone^21^.

Another advantage of using structural data for the individual networks is that the DTI scans are acquired non-invasively, unlike some studies that used functional networks based on invasive EEG recordings^24–26,33^. Similarly, no seizure activity or sleep deprivation is required, whereas some previous studies relied on seizure activity for their model^24–26,30,33^. However, recordings of interictal functional or structural activity are more patient-friendly, have fewer side-effects and are easier to acquire. Accordingly, methods that perform similarly using interictal data compared to ictal data^31^ are preferable to those that perform best using ictal data^26^.

The model was individualized for each patient, which is a hallmark of precision medicine. The varying results for different patients underline the need for using personalized modeling of seizure propagation. Other studies reported considerable variability among patients and epochs^26^ or recommended the use of individual structural networks^28^. One study compared the use of an average structural network with individual networks and found that virtual resections were more effective in stopping seizure propagation in their model with the individual networks^27^. It is therefore important to use individual networks when optimizing virtual resections.

## Limitations

The presented model is simple with very few parameters, but it does not capture the underlying biological basis. Instead, it captures one behavior that we want to study: the propagation of seizure activity. The seizure starts locally (few nodes in the model are active), propagates to connected areas (the number of active nodes increases), and eventually subsides (no more active nodes) (see Supplementary Figure S.1). A model should capture the behavior to be studied, but does not need to replicate the underlying biology truthfully or even approximate it^74,75^. In fact, a close match on details might even lead to false conclusions^33,76^. Thus, a model can capture the behavior in question without being true to the underlying biological basis.

Despite the simplicity of the model, there were still some parameters that needed to be set ad-hoc. In particular, to measure seizure spreading we set t_0_ = 10 in the SIR model. This value was selected to set the SIR model to the early phase, dominated by the propagation process, allowing us to model how much and how fast the seizure spreads, which can already be seen in the first steps of propagation. Moreover, for a given network, the maximum of I(t), indicating the severity of the epidemic (or seizure) is determined by the initial spreading^51^. Thus, we selected a small number of steps where the epidemic is dominated by spreading processes but enough time has passed so that a significant fraction of the network has been infected (since the SIR model was set well into the supercritical regime for each patient). However, the actual time considered here (t_0_ = 10) is arbitrary and, importantly, other values could be selected without affecting the qualitative results of the analysis.

Validation of the model is difficult, as we were not able to compare the suggested smaller resection to any actual treatment, because all connections were severed in the actual surgery. Some of the similar model studies described above circumvented this problem by comparing the propagation pathways of the simulated activity in the model to the propagation pathways of seizure activity in SEEG recordings^27,28^. Hutchings and colleagues^23^ validated their model by comparing the model findings in left TLE patients to those in healthy controls: patients transited from a non-epileptogenic state to an epileptogenic state more often than controls, and the most frequent starting point for seizures in patients was the left hemisphere^23^. Another possibility is to not use the information about the resection area or clinical SOZ for the model simulations, but for validation of the results. For example, Sinha et al. identified epileptogenic nodes in the model that overlapped with the clinically identified SOZ nodes^29^. The virtual resection of those epileptogenic nodes reduced the overall seizure likelihood in the model. Other model studies used surgical outcome for validation^25, 26,30,33^. We aimed to validate our model by using surgical outcome, such that the model results would differ between NSF and SF patients. We found that the NSF group had a (non-significant) larger EC of the seed, which consequently (and trivially) led to a marginally larger EC difference of the optimal resection, but the difference was not significant. The other studies that used validation found a large effect for SF patients and small effect for NSF patients^30,33^ or a positive correlation between effect and surgical outcome^25,26^. However, using surgical outcome is a start for validation, as the goal is to improve the surgical outcome. This underlines the need for a ground truth, which is inherently lacking in clinical research^77^.

## Outlooks

Such computer models as the one we presented here are the first step on a promising path: to create an individualized computer model that informs epilepsy surgery and thereby improves the outcome. The next milestone on this path is the prospective testing of such a model, where the model predictions made before the actual surgery are compared to the actual surgery and its outcome. Before such a step is taken, however, the model needs to be validated in a retrospective study and show a high accuracy that can be reproduced in an independent dataset. The validation is a challenge as the ground truth is inherently missing in clinical research. One possibility to approximate the ground truth of removing the areas or connections suggested by the model could be to temporarily inhibit the areas using brain stimulation, such as intermittent transcranial magnetic stimulation (iTMS) or transcranial direct/alternating current stimulation (tDCS/tACS). This validation would be limited to the inhibition of certain locations and area sizes, but it could reflect the effect of some of the virtual resection strategies, although the effects of excitation/inhibition in a network are not straightforward to predict^78^.

Alternative approaches to find the optimal connections for removal can be found in the extensive theoretical literature about the SIR model. For example, the link with the highest product of the two adjoining node’s EC can be removed as it increases the network’s epidemic threshold, below which activity does not propagate^79^. Otherwise, a centrality measure that is more closely related to information flow can be employed: the pseudoinverse of the Laplacian reflects how well a node spreads information^54^.

Future models could be extended to indicate the percentage of improvement for different resection strategies, instead of giving a binary suggestion (recommended or not recommended). In this way, the risk and benefits of certain resection strategies could be balanced against each other. Furthermore, no-go areas can be incorporated that are blocked for removal. These blocked connections or areas could represent eloquent cortex, but also the practical implementation of certain strategies. For example, connections or areas might be grouped together and marked for resection as they are spatially close, or it might be impractical to resect one area and disconnect a remote connection at the same time. Additionally, here we only considered connections from the hypothesized EZ as candidates for resection. Including all network connections may result in more effective resections. Finally, nodes could be removed in the model instead of connections.

## Conclusion

Tailored computational models that take into account the patient-specific connectivity might be the next step to improve the outcome of epilepsy surgery. Before they can be applied in clinical practice, such models need to be extensively validated via retrospective and prospective studies. In this study, we used a spreading model together with a surrogate measure to define a simple computational model to perform virtual resections on individual structural brain networks. The goal was to reduce seizure propagation in the model and thereby inform epilepsy surgery about the optimal strategy. Using the surrogate measure, a smaller resection achieved almost the same effect as the actual surgery, at a considerably smaller cost. The connections that were predominantly removed were those connecting the EZ to hub nodes, whereas connections that were less important for seizure propagation could be spared. A more limited resection could mean fewer side-effects and cognitive complaints after surgery, thereby improving the quality of life of the patient.

Computer models can aid epilepsy surgery by tailoring the resection area, testing competing resection strategies, and finding alternative resections when eloquent cortex overlaps with the seizure onset zone. Beyond epilepsy surgery, computer models can inform other types of neurosurgery (e.g. brain tumor surgery), help unravel the mechanism of epilepsy or other diseases (e.g. spread of tau pathology) and shed light on the general workings of the brain (e.g. how structure and function interrelate).

## Supplementary Information

Supplementary Information.

## Data Availability

The raw patient data cannot be shared as the patients did not consent to data sharing. Metadata and code are available upon reasonable request to the corresponding author under the condition of an existing collaboration agreement.
