# Supplementary material for: Optimization of epilepsy surgery through virtual resections on individual structural brain networks
Source: Sci Rep. 2021 Sep 24;11:19025. doi: 10.1038/s41598-021-98046-0 (PMC8463605; doi:10.1038/s41598-021-98046-0)
Supplement: Supplementary file 1 — Supplementary Information. [file 41598_2021_98046_MOESM1_ESM.pdf]

Supplementary Information for "Optimization of epilepsy surgery through virtual resections on individual structural brain networks"

Ida A. Nissen[1], Ana P. Millán[1], Cornelis J. Stam[1], Elisabeth C.W. van Straaten[1], Linda Douw[2], Petra J.W. Pouwels[3], Sander Idema[4], Johannes C. Baayen[4], Demetrios Velis[1], Piet Van Mieghem[5], and Arjan Hillebrand[1]

[1]Amsterdam UMC, Vrije Universiteit Amsterdam, Department of Clinical Neurophysiology and MEG Center, Amsterdam Neuroscience, De Boelelaan 1117, Amsterdam, The Netherlands.
[2]Department of Anatomy and Neuroscience, Amsterdam Neuroscience, Vrije Universiteit Amsterdam, Amsterdam UMC, Amsterdam, The Netherlands
[3]Radiology and Nuclear Medicine, Amsterdam Neuroscience, Vrije Universiteit Amsterdam, Amsterdam UMC, Amsterdam, The Netherlands
[4]Department of Neurosurgery, Amsterdam Neuroscience, Vrije Universiteit Amsterdam, Amsterdam UMC, Amsterdam, The Netherlands
[5]Faculty of Electrical Engineering, Mathematics and Computer Science, Delft University of Technology, Delft, The Netherlands

1 The SIR model on a brain network

Supplementary Figure $S.1$ illustrates the SIR model on a brain network.

2 Link between the Eigencentrality and SIR propagation

2.1 Principal Eigenvector of the Adjacency Matrix and Epidemic Spreading

A network of N nodes is typically represented by its adjacency matrix A, which is a symmetric $N \times N$ matrix, where element $a_{ij} = 1$ if node i and node j are connected by a link, and $a_{ij} = 0$ otherwise. Another representation of the network is given by the associated symmetric Laplacian Q. A key property of symmetric matrices [1] is that their eigenvectors are real and orthogonal vectors. Thus, the set of eigenvectors constitutes a basis of the $N-$dimensional space. This means that any network state vector $s(t) = (s_1(t), s_2(t), ..., s_N(t))$, where $s_i(t)$ is the state of node i of the network, can be written as a linear combination of the eigenvectors of both the adjacency matrix A or the Laplacian Q.

The eigenvectors x_j of the adjacency matrix A satisfy the eigenvalue equation $Ax_j = \lambda_j x_j$, where λ_j is the real eigenvalue corresponding to the eigenvector x_j. Any network state vector $s(t)$ can be represented by

$$s(t) = \sum_{i=1}^{N} \alpha_i(t) x_i, \tag{1}$$

where the coordinate $\alpha_i(t) = s^T(t) x_i$ is the projection of the state vector $s(t)$ onto the eigenvector x_i. By the Perron-Frobenius theorem [1] for non-negative matrices (such as the adjacency matrix A but not

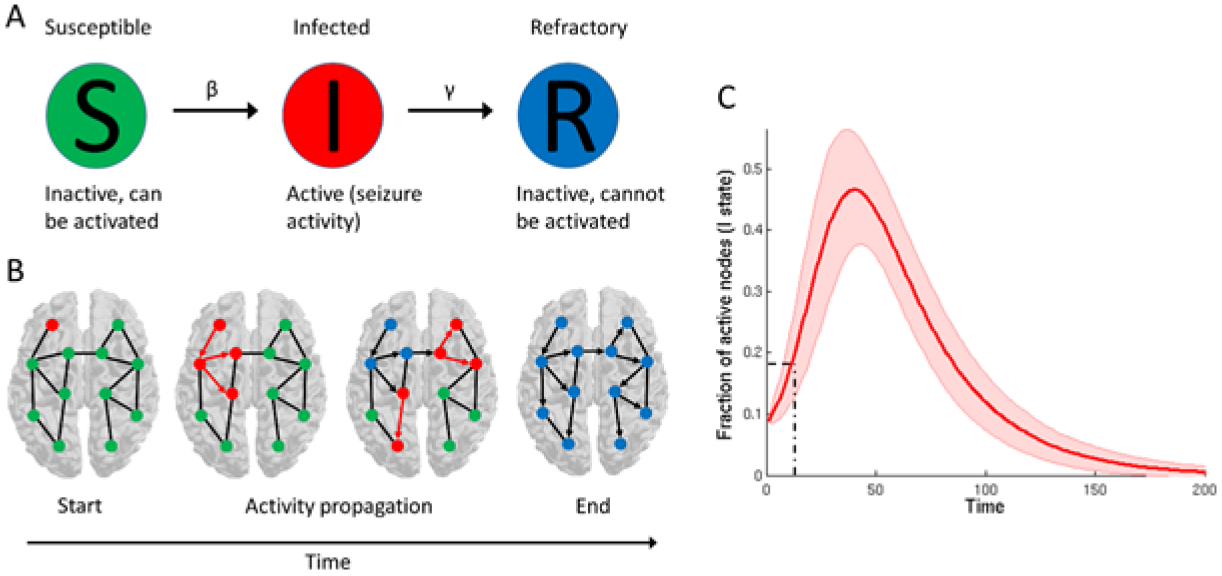

Supplementary Figure S.1: The SIR model on a brain network. (A) Each node in the network can be in one of three states: S (susceptible – node is inactive and can be activated), I (infected – node is active/seizing), and R (refractory – node is inactive and remains inactive). The probabilities of transitioning to the next state are given by beta (β) and gamma (γ). (B) Initially, only the nodes of the hypothesized EZ (here: left frontal) are active. The activity propagates to each neighbouring node with probability β at each time step. Additionally, an active node can turn refractory with probability γ at each time step. At the end, all nodes are either refractory or susceptible. (C) The percentage of active nodes (I) is shown over time of an example simulation (patient 9). The activity propagates quickly from the hypothesized EZ to the rest of the brain. After reaching a maximum, the fraction of active nodes decreases, as more nodes turn refractory, until it reaches zero. We used the fraction of active nodes at time step $t = 10$ to quantify the speed of propagation ($I_{t=10}$).

the Laplacian Q) the largest eigenvector x_1 possesses all non-negative components. Thus, in a connected graph each component $(x_1)_m$ of the principal eigenvector $x_1 = ((x_1)_1, (x_1)_2, ..., (x_1)_N)$ is positive.

Let $s(t)$ represent the probability vector that each node is infected in the epidemic process. Then, the projection $\alpha_1(t) = s^T(t)x_1 = \sum_{k=1}^{N} s_k(t)(x_1)_k$ is the largest, because all terms in the sum are positive. Any other eigenvector must have negative components, due to the orthogonality condition ($x_i^T x_j = \delta_{i,j}$ for any pair (y_i, y_j)). In other words, the state vector $s(t)$ is most aligned with the principal (largest) eigenvector of the adjacency matrix, which means that

$$s(t) = \alpha_1(t)x_1 + r(t), \tag{2}$$

where $r(t) = \sum_{i=2}^{N} \alpha_i(t)x_i$ can be regarded as a correction. In particular, in a mean-field setting the viral state vector $s(t)$ is proportional to the eigenvector x_1 when the epidemic process is very close to and above the epidemic threshold and, consequently, the correction $r(t)$ is very small [2, 3]. The further the epidemic process operates above the epidemic threshold, the more other eigenvector components must be incorporated in the correction $r(t)$.

The eigenvectors of the adjacency matrix A of a graph appear in many other dynamic processes on networks, and particularly, in synchronization processes [4, 5]. Surprisingly, little is known about their "physical" meaning. A relevant interpretation follows from the stochastic matrix $P = \Delta^{-1}A$, where $\Delta = \text{diag}\,(d_j)$ and $d_j = \sum_{i=1}^{N} a_{ij}$. P represents the discrete Markov probability transfer matrix characterizing a random walk on the graph G [1]. The jth element of the largest eigenvector x_1 of P, $x_1^{[j]}$ (normalized as $u^T x_1 = 1$, where u is the all one vector), reflects the probability that a random walk on the graph G visits node j. In particular, the number $N_k(j)$ of walks of length k starting at node j is given by [6]

$$\lim_{k\to\infty} \frac{N_k(j)}{\sum_{j=1}^{N} N_k(j)} = x_1^{[j]}. \tag{3}$$

In a discrete-time setting as our scenario, the length k of a walk equals the discrete time k, assuming the random walk started at time 0. Thus, relation (3) indicates that the fraction of time that the ever-lasting random walk visits node j equals the principal eigenvector component of the adjacency matrix.

Random walks have been extensively related to spreading processes [7, 8] and to the spectral properties of a network [**millan2020complex**, 9]. In particular, in Ref. [8] it was shown that the basic voltage-current relation (better known as the law of Ohm) in a resistor network is $x = \tilde{Q}v$, where $\tilde{Q}$ is the $N \times N$ weighted Laplacian matrix, x is the current vector and v is the potential vector. Thus, v_j is the potential at node j and x_m the electrical current injected in node m. Denoting the pseudoinverse of the weighted Laplacian as $\tilde{Q}^\dagger$, $v = \tilde{Q}^\dagger x$ represents the nodal potential as function of the injected current (provided the reference potential is chosen equal to the average voltage $\frac{1}{N}v^T u$). The vector $\zeta = \left(\tilde{Q}^\dagger_{11}, \tilde{Q}^\dagger_{22}, ..., \tilde{Q}^\dagger_{NN}\right)$ consisting of the diagonal elements of $\tilde{Q}^\dagger$, is a graph metric vector with the same importance as the degree vector $d = (Q_{11}, Q_{22}, ..., Q_{NN})$. As shown in Ref. [8], the smallest vector component k of ζ, that minimizes $\tilde{Q}^\dagger_{kk} \le \tilde{Q}^\dagger_{jj}$ for $1 \le j \le N$, can be regarded as the best spreader node in the graph or as the node lying at the center of gravity of the graph. Thus, ranking the components of the vector ζ also ranks the nodes with respect to their spreading capability. For different graphs, the correlation between the principal eigenvector x_1 and the vector ζ as well as the projection $\zeta^T x_1$, has been shown to correlate strongly [8].

2.2 Numerical validation

In figure 7 of the main text we showed that the EC of a node strongly correlated with $I(t_0)$ when the node is used as the single seed for seizure propagation. In order to validate whether this relationship holds also when the seed includes more than one node, we considered the full resection area as the seed for propagation, and compared $I(t_0)$ with the EC of the seed, across patients, in figure $S.2$. To control for the different sizes among the seeds, we represented the total EC of the seed (instead of the average

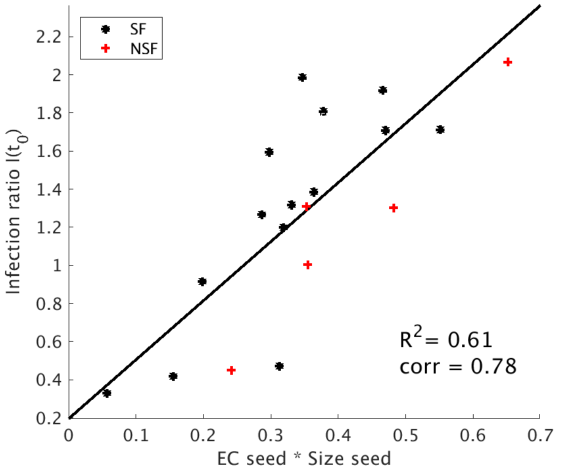

Supplementary Figure S.2: Correlation of the nodal EC with the SIR model across patients. The seed was defined as the full resection area for each patient, and the EC of the seed was calculated as the sum of nodal EC of each node in the seed. We then simulated the SIR model independently for each patient, and measured $I(t_0)$, for $t_0 = 10$, averaged over 10^4 runs. The correlation was 0.78. SF and NSF are indicated by black stars and red crosses, respectively. All patients were included in the correlation analysis.

value). We found a strong correlation between the total EC of the seed and $I(t_0)$ ($C = 0.78$), despite the fact that each data-point corresponds to a different network. Thus, figure $S.2$ indicates that the relation between the EC of the seed and $I(t_0)$ still holds when the seed includes more than one node.

3 Average Networks

In order to establish the effect of the patient's individual connectivity (B), we have repeated the analysis on the average DTI matrix (AV). This has been constructed by averaging the weighted individual DTIs and subsequently thresholding them at 0.11 density, as in the main text. The EC decrease as a function of the number of removed links is shown in the top left panel of Supplementary Figure $S.3$.

We compared the two models according to the number of links from the SOZ to the rest of the network (L), the number of removed edges to obtain a 90% EC reduction, the fraction of removed links (FRL) and the EC difference of both the full and the 90% resections (respectively EC Diff and EC DIff (90%)). For the comparison we used paired Student T-tests. The details are indicated in table S.1. An individual comparison of the number of links and the EC difference between B and AV networks is shown in Supplementary Figure $S.3$ (respectively top right and bottom left panels).

We found L to be larger in the individual networks ($\mathrm{L}_B - \mathrm{L}_{AV} = 1.95$, $p = 0.02$). RL was also smaller for the average network, but the difference is not significant ($\mathrm{RL}_B - \mathrm{RL}_{AV} = 1.00$, $p = 0.08$), and the fraction of removed edges ($FRL = RL/L$) was comparable ($\mathrm{FRL}_B - \mathrm{FRL}_{AV} = -0.01$, $p = 0.51$). Similarly, the EC difference was not significantly different between the models ($\mathrm{EC\ Diff}_B - \mathrm{EC\ Diff}_{AV} = -0.0045$, $p = 0.20$).

Finally, we compared the overlap between the 90% resections (see the bottom right panel of Supplementary Figure $S.3$). On average, the AV resection covered 81.06% of the B one, while the B resection covered 88.43% of the AV one. The average intersection between the resections was 61.78%.

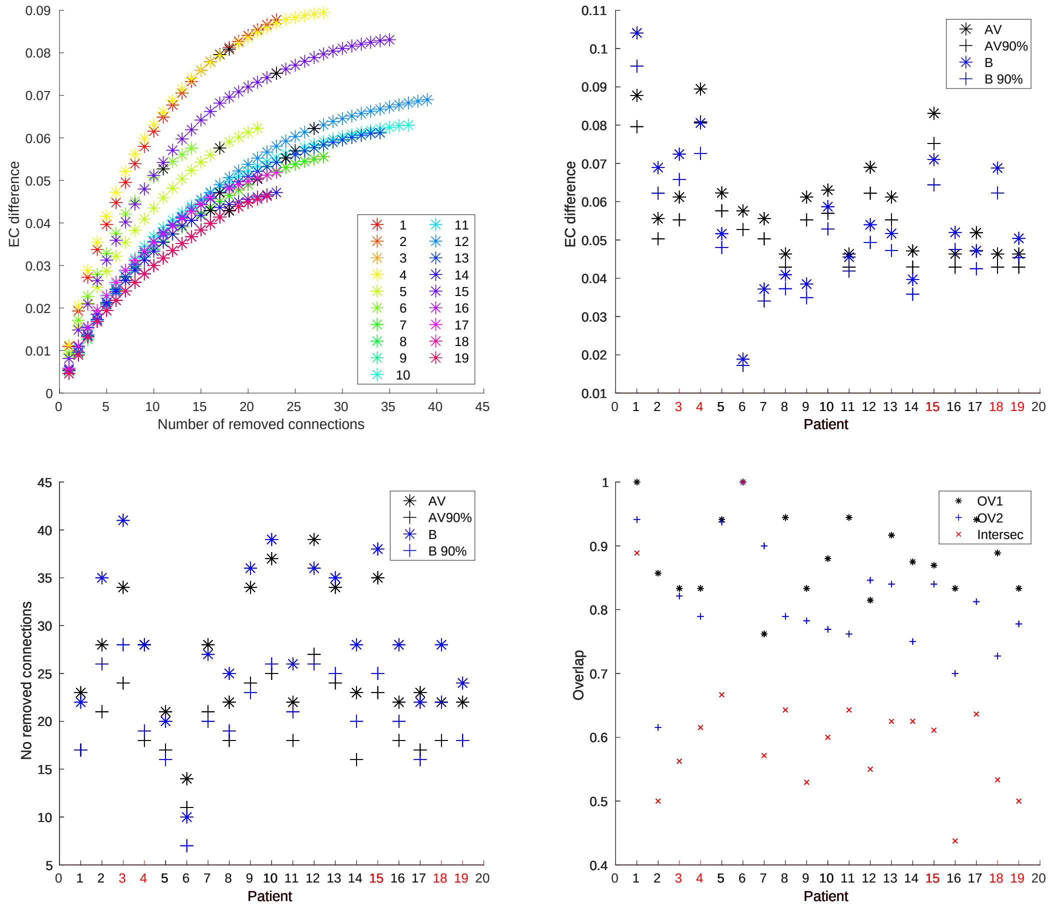

Supplementary Figure S.3: Comparison with the AV network. The top left panel shows the EC difference as a function of the number of removed links, for each patient. Black stars indicate the 90% reduction. The top right shows the total (stars) and the 90% (crosses) EC difference for the averaged (AV) (black markers) and individual (B) (blue markers) networks, for each patient. Similarly, the bottom left panel shows the number of links from the SOZ (stars) and the number of resected links for the 90% EC decrease (crosses), for the AV (black markers) and B (blue markers) networks, for each patient. Finally, the bottom right panel indicates the overlap between the B and AV resections, for each patient. The black stars (blue crosses) show OV1 (OV2), the portion of the AV (B) resection covered by the B (AV) one. The red markers indicate the intersection between the resections. In the last three panels we indicate with red labels the patients who where not seizure free.

	L	RL	FRL	EC Diff	EC Diff (90%)
B	28.84 ± 7.79	20.74 ± 4.95	0.72 ± 0.08	0.0554 ± 0.019	0.0504 ± 0.0123
AV	26.89 ± 6.81	19.74 ± 3.94	0.73 ± 0.06	0.0599 ± 0.0139	0.0545 ± 0.0123
Diff	1.95	1.00	-0.01	-0.0045	-0.0042
CI	(3.55, 0.35)	(2.11, -0.11)	(0.00, -0.04)	(0.0026, -0.0116)	(0.0022, -0.0105)
(t, p)	(2.55, 0.02)	(1.89, 0.08)	(-2.09, 0.51)	(-1.34, 0.20)	(-1.38, 0.19)

Supplementary Table S.1: Comparison between the individual patient networks (B) and the averaged (AV) ones. We indicate the number of links L, the number of resected links RL, the fraction of resected links FRL and the EC differences corresponding to the full (EC Diff) and the 90% (EC Diff 90%) resections, as indicated by the label of each column. The two first rows indicate the average values for the individual (B) and averaged (AV) networks, with an error given by the standard deviation. The last three rows indicate the difference (B-AV), confidence interval and t, p pairs from the Student T-tests. The number of degrees of freedom is 18 in all cases.

	L	RL	FRL	EC Diff	EC Diff (90%)
B	28.84 ± 7.79	20.77 ± 4.95	0.72 ± 0.05	0.0554 ± 0.0191	0.0504 ± 0.0173
W	312.68 ± 109.59	11.47 ± 5.47	0.036 ± 0.013	0.0272 ± 0.0688	0.0260 ± 0.0680
Diff	-284	9.26	0.68	0.0282	0.0243
CI	(-233, -334)	(10.99, 7.53)	(0.71, 0.67)	(0.0661, -0.0097)	(0.0613, -0.0127)
(t, p)	(-11.87, $6 \cdot 10^{-10}$)	(11.25, $1.4 \cdot 10^{-9}$)	(60.55, $3 \cdot 10^{-22}$)	(1.56, 0.14)	(1.38, 0.18)

Supplementary Table S.2: Comparison between the binary (B) and weighted (W) patient networks. This table is structured as table $S.1$: each column indicates respectively the number of links L, the number of resected links RL, the fraction of resected links FRL and the EC differences corresponding to a full (EC Diff) and 90% (EC Diff 90%) resections. The two first rows indicate the average values respectively for the B and W networks, with an error given by the standard deviation. The last three rows indicate the difference (B-W), confidence interval and t, p pairs from the Student T-tests. The number of degrees of freedom is 18 in all cases.

4 Weighted Networks

In order to establish the effect of thresholding and binearizing the connectivity matrices, we repeated the analysis on the individual weighted DTI matrices (W). We followed the same procedure as in the previous section; the details are indicated in table $S.2$.

Given that the W matrices are not thresholded, the number of links was significantly larger than in the binarized matrices (B) ($\mathrm{L}_B - \mathrm{L}_W = -284$, $p < 10^{-4}$). Due to the rapid decrease in weight strength, however, the 90% resection was smaller ($\mathrm{RL}_B - \mathrm{RL}_W = 9.26$, $p < 10^{-4}$). The EC values were also affected by the weights, and the EC difference was smaller for the W networks ($\mathrm{EC\ Diff}_B - \mathrm{EC\ Diff}_W = 0.028$), but the difference was not significant ($p = 0.14$).

Finally, we compared the overlap between the 90% resections. On average, the W resection covered 60.94% of the B one, while the B resection covered 83.44% of the W one. The average intersection between the resections was 43.09%.

Because the DTI weights are based on fiber-tracking probabilities, and the may underestimate the contribution of weaker links, we repeated the analysis on the logarithmic version of the networks, logW, with components $logW_{ij} = \log_{10}(w_{ij}/w_m)$ if $w_{ij} > 0$, and 0 otherwise. The normalizing factor w_m is the smallest positive (i.e. greater than 0) entry of W, and it is used to avoid negative values in logW. We performed the same analysis as in the previous sections; the details are indicated in table $S.3$.

We found that with this metric the resection needed to achieve a 90% reduction was much bigger than with the binary networks ($\mathrm{RL}_B - \mathrm{RL}_{logW} = -183$, $p < 10^{-4}$), but the fraction of removed links was

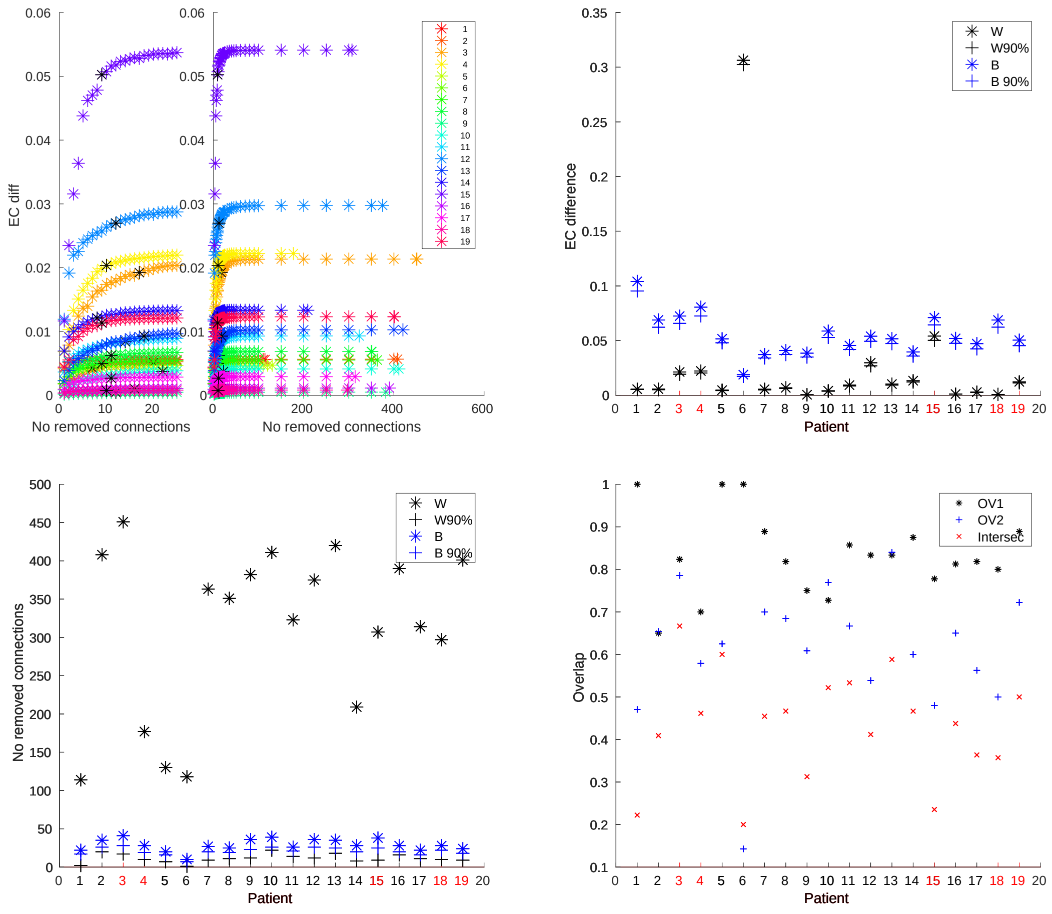

Supplementary Figure S.4: **Comparison with weighted networks**. The top left panel shows the EC difference as a function of the number of removed links, for each patient. Black stars indicate the 90% reduction. On the left plot we show a zoom in for few removed connections, where the 90% resection is found. The other three panels indicate the EC difference, number of links and overlap for each patient, as in Supplementary Figure $S.3$.

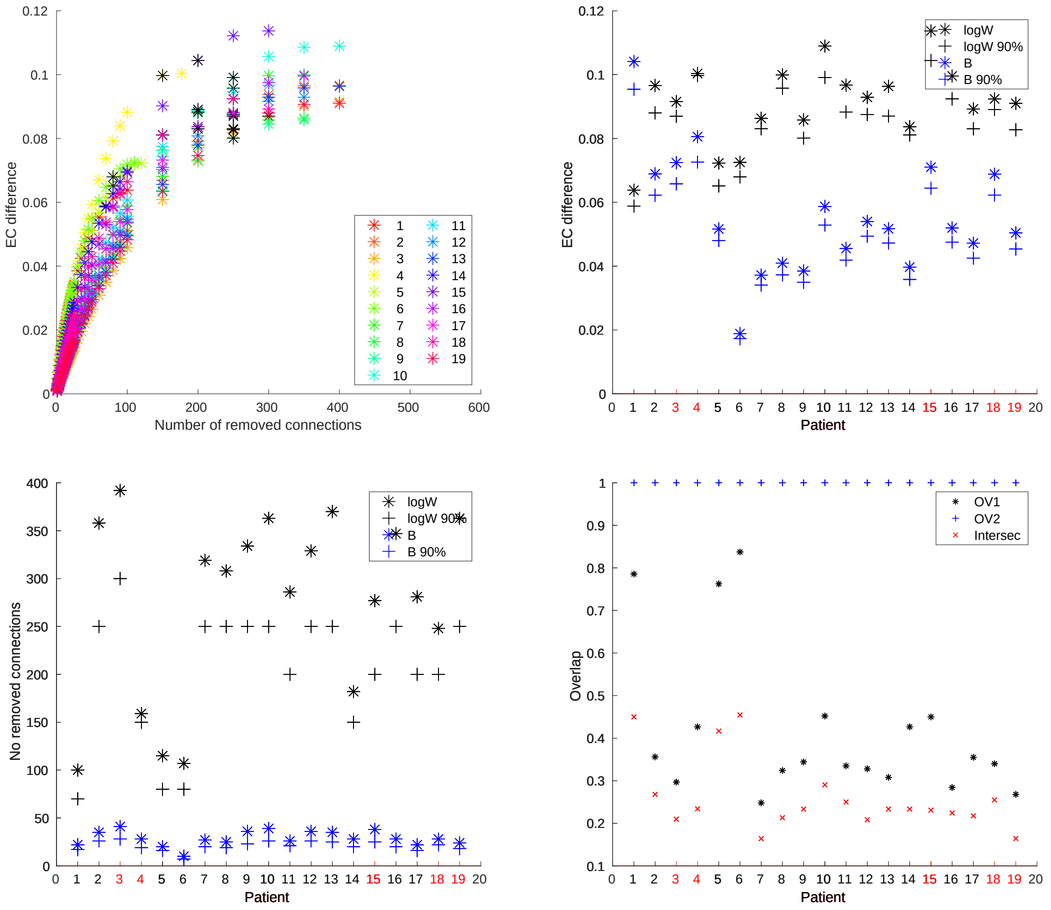

Supplementary Figure S.5: **Comparison with log-scale weighted networks.** The top left panel shows the EC difference as a function of the number of removed links, for each patient. Black stars indicate the 90% reduction. On the left plot we show a zoom in for few removed connections, where the 90% resection is found. The other three panels indicate the EC difference, number of links and overlap for each patient, as in Supplementary Figure $S.3$.

similar ($\mathrm{FRL}_B - \mathrm{FRL}_{logW} = -0.02$, $p = 0.29$). The total EC difference was also significantly larger for logW networks ($\mathrm{EC\ Diff}_B - \mathrm{EC\ Diff}_{logW} = -0.0359$, $p < 10^{-4}$) due to the change in weight scale.

We found that the logW resection completely covered the B one (100%), whereas the B resection covered 41.72% of the logW one. The average intersection between the resections was 26.05%.

References

[1] P. Van Mieghem. *Graph spectra for complex networks*. Cambridge University Press, 2010.

[2] P. Van Mieghem, J. Omic, and R. Kooij. "Virus spread in networks". In: *IEEE/ACM Transactions On Networking* 17.1 (2008), pp. 1–14.

[3] B. Prasse and P. Van Mieghem. "Time-dependent solution of the NIMFA equations around the epidemic threshold". In: *Journal of Mathematical Biology* 81.6 (2020), pp. 1299–1355.

	L	RL	FRL	EC Diff	EC Diff (90%)
B	28.84 ± 7.79	20.74 ± 4.95	0.72 ± 0.05	0.0554 ± 0.0191	0.0504 ± 0.0173
logW	276 ± 96	204 ± 68	0.74 ± 0.07	0.0912 ± 0.0123	0.0853 ± 0.0116
Diff	-246.84	-183.48	-0.02	-0.0359	-0.0349
CI	$-202,\ -290$	$-152,\ -214$	$0.02,\ -0.06$	$-0.0252,\ -0.0465$	$-0.0251,\ -0.0448$
$t,\ p$	$-11.81,\ 6 \cdot 10^{-10}$	$-12.43,\ 3 \cdot 10^{-10}$	$-1.08,\ 0.3$	$-7.08,\ 1.3 \cdot 10^{-6}$	$-7.45,\ 7 \cdot 10^{-7}$

Supplementary Table S.3: Comparison between the binary (B) and log-weighted (logW) patient networks. Each column indicates respectively the number of links L, the number of resected links RL, the fraction of resected links FRL and the EC differences corresponding to a full (EC Diff) and 90% (EC Diff 90%) resections. The two first rows indicate the measured values for each case, whereas the last three rows indicate the difference (B-W), confidence interval and t, p pairs from the Student T-tests. The number of degrees of freedom is 18 in all cases.

[4] P. Van Mieghem. “Graph eigenvectors, fundamental weights and centrality metrics for nodes in networks”. In: *arXiv preprint arXiv:1401.4580* (2014).

[5] P. Van Mieghem. “Epidemic phase transition of the SIS type in networks”. In: *EPL (Europhysics Letters)* 97.4 (2012), p. 48004.

[6] D. Cvetkovic et al. *Eigenspaces of graphs*. 66. Cambridge University Press, 1997.

[7] P. G. Doyle and J. L. Snell. *Random walks and electric networks*. Vol. 22. American Mathematical Soc., 1984.

[8] P. Van Mieghem, K. Devriendt, and H. Cetinay. “Pseudoinverse of the Laplacian and best spreader node in a network”. In: *Physical Review E* 96.3 (2017), p. 032311.

[9] A. P. Millán et al. “Local topological moves determine global diffusion properties of hyperbolic higher-order networks”. In: *arXiv preprint arXiv:2102.12885* (2021).
